# Phospholipid metabolism-related genotypes of PLA2R1 and CERS4 contribute to nonobese MASLD

**DOI:** 10.1097/HC9.0000000000000388

**Published:** 2024-06-05

**Authors:** Congxiang Shao, Junzhao Ye, Zhi Dong, Bing Liao, Shiting Feng, Shixian Hu, Bihui Zhong

**Affiliations:** 1 Department of Gastroenterology of the First Affiliated Hospital, Sun Yat-sen University, Guangzhou, China; 2 Department of Radiology of the First Affiliated Hospital, Sun Yat-sen University, Guangzhou, China; 3 Department of Pathology of the First Affiliated Hospital, Sun Yat-sen University, Guangzhou, China; 4 Department of Precision Medicine, Institute of Precision Medicine, The First Affiliated Hospital, Sun Yat-Sen University, Guangzhou, Guangdong, China

## Abstract

**Background:**

Abnormal phospholipid metabolism is linked to metabolic dysfunction–associated steatotic liver disease (MASLD) development and progression. We aimed to clarify whether genetic variants of phospholipid metabolism modify these relationships.

**Methods:**

This case-control study consecutively recruited 600 patients who underwent MRI-based proton density fat fraction examination (240 participants with serum metabonomics analysis, 128 biopsy-proven cases) as 3 groups: healthy control, nonobese MASLD, and obese MASLD, (n = 200 cases each). Ten variants of phospholipid metabolism-related genes [phospholipase A2 Group VII rs1805018, rs76863441, rs1421378, and rs1051931; phospholipase A2 receptor 1 (PLA2R1) rs35771982, rs3828323, and rs3749117; paraoxonase-1 rs662 and rs854560; and ceramide synthase 4 (CERS4) rs17160348)] were genotyped using SNaPshot.

**Results:**

The T-allele of CERS4 rs17160348 was associated with a higher risk of both obese and nonobese MASLD (OR: 1.95, 95% CI: 1.20–3.15; OR: 1.76, 95% CI: 1.08–2.86, respectively). PLA2R1 rs35771982-allele is a risk factor for nonobese MASLD (OR: 1.66, 95% CI: 1.11–1.24), moderate-to-severe steatosis (OR: 3.24, 95% CI: 1.96–6.22), and steatohepatitis (OR: 2.61, 95% CI: 1.15–3.87), while the paraoxonase-1 rs854560 T-allele (OR: 0.50, 95% CI: 0.26–0.97) and PLA2R1 rs3749117 C-allele (OR: 1.70, 95% CI: 1.14–2.52) are closely related to obese MASLD. After adjusting for sphingomyelin level, the effect of the PLA2R1 rs35771982CC allele on MASLD was attenuated. Furthermore, similar effects on the association between the CERS4 rs17160348 C allele and MASLD were observed for phosphatidylcholine, phosphatidic acid, sphingomyelin, and phosphatidylinositol.

**Conclusions:**

The mutations in PLA2R1 rs35771982 and CERS4 rs17160348 presented detrimental impact on the risk of occurrence and disease severity in nonobese MASLD through altered phospholipid metabolism.

## INTRODUCTION

Metabolic dysfunction–associated steatotic liver disease (MASLD), redefined as NAFLD, is a worrisome public health burden with a worldwide prevalence of 29.8% and continues to predict a growing incidence.^1^,^2^ The surging MASLD epidemic has been widely attributed to a parallel increase in the prevalence of obesity. However, MASLD also occurs in nonobese individuals. The estimated prevalence of nonobese MASLD ranges widely, from 7% to 20% in Western populations (19.3% in North America; 95% CI: 13.9–26.2) and 5%–26% in Asian population.^3^ Furthermore, it has been reported that patients who are not obese with MASLD had a relatively milder metabolic abnormality and histological changes than the obese ones but a similar cardiovascular disease risk.^4^,^5^,^6^ Therefore, the genetic risk factor may play important roles in the presence and progression of MASLD.

Genetic predisposition to MASLD has been widely demonstrated in other studies, with patatin-like phospholipase domain-containing protein 3 rs738409 being the genetic variant most strongly associated with MASLD.^7^ In addition, sterol regulatory element‐binding factor, transmembrane 6 superfamily member 2, cholesteryl ester transfer protein, and apolipoprotein C3 influence the development and presence of nonobese MASLD.^8^,^9^,^10^,^11^ Examining other potential genetic variations associated with lipid or steatosis homeostasis can help identify patients at a higher risk of MASLD at an early stage and understand their clinical significance.

The primary components of the human cell membrane are phospholipids, specifically phosphatidylcholine (PC) and phosphatidylethanolamine (PE).^12^ Spatial mass spectrometry imaging with lipidomics for both high-fat-induced and ob/ob MASLD models illustrated specific phospholipid remodeling with distinct zonal distributions among the liver tissues of control, simple fatty liver, and steatohepatitis. It was identified that docosahexaenoic acid-containing PC(38:6), PC(40:7), and PC(40:6) aggregated more in zone 3 of the liver parenchyma.^12^ In addition, increased hepatic expression of lysophosphatidylcholine acyltransferase 2 and cytoplasmic phospholipase A2 may account for the observed phospholipid membrane remodeling in steatohepatitis. Animal experiments revealed that the PC/phosphatidylethanolamine ratio is a crucial regulator of cell membrane integrity and is involved in the progression of simple steatosis to steatohepatitis.^13^ These studies indicate that changes in phospholipid components and related metabolic pathways are associated with MASLD. Phospholipase A2 group VII, phospholipase A2 receptor 1 (PLA2R1), paraoxonase-1 (PON1), ceramide synthase 4 (CERS4) are crucial molecules in the regulation of phospholipid metabolism. Mutations in these genes (phospholipase A2 group VII rs1805018, rs76863441, rs1421378, and rs1051931; PLA2R1 rs35771982, rs3828323, and rs3749117; PON1 rs662, rs854560; and CERS4 rs17160348) were found to be risk factors for cardiovascular events or influence the serum level of Lp-PLA2.^14^,^15^,^16^ However, whether these gene polymorphisms confer a higher risk for MASLD remains unclear.

This study aimed to investigate the association between phospholipid metabolism-related gene polymorphisms and susceptibility to MASLD. Furthermore, we explored potential gene polymorphisms associated with alterations in serum phospholipid metabolite levels. Additionally, we investigated factors associated with moderate-to-severe steatosis, carotid plaque, steatohepatitis, and significant fibrosis.

## METHODS

### Study population

This was a prospective case-control study conducted at the First Affiliated Hospital, Sun Yat-Sen University. The study protocol was approved by the ethics committee of the First Affiliated Hospital, Sun Yat-Sen University (Approval number: [2020]187), registered at the Chinese Clinical Trial Registry, ChiCTR-ChiCTR2000034197, https://www.chictr.org.cn/) and was performed in accordance with the ethical standards of the 1964 Declaration of Helsinki. All the patients signed the informed consent. Consecutive participants with liver fat contents (LFC) measurements using MRI-proton density fat fraction were recruited from January 2017 to October 2020 and their diagnosis has been further confirmed according to the MASLD definition. Hepatic steatosis was confirmed as the LFC ≥ 5%, while the individuals with LFC < 5% and traceable negative medical history information were classified as healthy controls.^17^,^18^ The body mass index (BMI) cutoff value of 23 kg/m^2^ was adopted to define the nonobese.^19^

All the enrollees were required to be at least 18 years old with completed anthropometric data and laboratory and imaging examination results. Apart from the presence of hepatic steatosis, the finding of any of a cardiometabolic risk factor (at least 1 out of 5), would confer a diagnosis of MASLD if there are no other causes of hepatic steatosis. The exclusion criteria included: over alcohol intake (≥ 140 g/week in female and ≥ 210 g/week in male), positive markers of viral infection such as HBsAg or antibody against HCV; autoimmune liver disease; endocrine disorders (eg, hypothyroidism, hypogonadism); competing etiologies of liver disease leading to steatosis (eg, consumption of tamoxifen and amiodarone); pregnancy and malignancies.^4^,^5^

### Clinical assessment

All participants provide self-reported alcohol consumption, smoking, medical history through a structured questionnaire. Anthropometric data (weight, height, waist circumstance, and hip circumference) were obtained by 2 fixed and trained physicians. Waist-to-hip ratio was calculated as the waist circumstance divided by hip circumference. After fasting for 8 hours, blood samples were drawn for the assessment of biochemistry (ALT, AST, uric acid [UA]), fasting insulin (FINS), fasting blood glucose (FBG) and lipids (total cholesterol (CHOL), triglycerides (TG), high-density lipoprotein-cholesterol (HDL-C), low-density lipoprotein-cholesterol [LDL-C]). The Homeostatic Model Assessment of Insulin Resistance (HOMA-IR) was calculated as FINS (μU/mL) × FBG (mmol/L)/22.5.^5^ A cutoff value of 2.5 was used to define insulin resistance (IR).^20^

### Quantification of liver fat content by MRI-proton density fat fraction

Upper-abdominal MRI with a 3.0-Tesla MRI scanner (Siemens 3.0T MAGNETOM Verio) was conducted within 2 weeks after laboratory measurements were finished. The scanning protocol and the sequence parameter settings follow other studies’s descriptions.^21^,^22^ The scanning protocol and imaging parameters were as our previous studies described: TE1 2.5 ms, TE2 3.7 ms, 5.47 ms for repetition; 5° flip angle; ± 504.0 kHz per pixel receiver bandwidth, and a slice thickness of 3.0 mm. LFC was estimated using the images of fat-water separation. We further classified fatty liver as mild (5.0%–16.3%), moderate (16.3%–21.7%) and severe (> 21.7%) according to the LFC cutoff values that had been validated clinical trials for evaluating the effectiveness of different medication therapy on MASLD.^23^

### Liver stiffness measurement by 2D-SWE

Liver stiffness measurement was performed through 2D-SWE during the initial clinical assessment after fasting overnight. Measurements were obtained by means of the intercostal spaces with the patients in the dorsal decubitus position and their right arm maximally abducted. The measurement was performed 1–2 cm under the liver capsule in an area of the parenchyma. Circular ROIs were placed in areas of homogeneous portion of the speed image and avoided vascular and biliary structures.^24^ The examination results were considered valid with at least 10 eligible acquisitions, success rate ≥ 60% and the interquartile range-to-median ratio < 0.3. A cutoff value of 7.1 kPa was used to define significant fibrosis.^25^

### Histological assessment

Liver biopsy was performed in the right hepatic lobe under ultrasound guidance using 18 G Temno needles. Two passes of liver specimens were attained for each patient to get the samples with at least 15 mm in length. The histological characteristics were evaluated and scored using the NASH clinical research network system and steatosis, activity, and fibrosis scoring system. Fibrosis was graded using the Kleiner Fibrosis Score (F0–F4).^26^,^27^ Two fixed pathologists with over 10 years of experience were blinded to all the clinical data and reviewed the liver specimens separately. For the inconsistency in scoring, a third pathologist would participate in the discussion to achieve a final consensus.

### Metabonomics

For ultrahigh-performance liquid chromatography coupled with quadrupole time-of-flight mass spectrometry, liquid chromatographic analysis was performed on the Waters ACQUITY UPLC Iclass system (Waters Ltd. USA) coupled with an ACQUITY UPLC BEH C18 column. The original data was imported into the Progenesis QI (Nonlinear Dynamics Waters, UK) software to extract the matched peak of filtering noise peak and EZinfo 3.0 for Waters (Umetrics, Sweden) software was used for further analysis. Phospholipid compound identification and analysis based on the phospholipid cleavage law and online databases METLIN metabolite MSMS database and LipidMaps. Detailed parameter settings were in accordance with our previous study descriptions.

For gas chromatography-mass spectrometry, an Agilent 7890 Guanine Cytosine (GC) system equipped with a 5977-quadrupole mass selective detector (Agilent, Santa Clara, CA) and an 5% phenyl methyl siloxane (HP-5MS) column (40 m × 0.25 mm inner diameter × 0.25 μm film thickness, Agilent, Santa Clara, CA) was used to obtain metabolic profiles of the derivatized products. Recorded mass spectra were compared with those stored in the National Institute of Standards and Technology US Government library. Detailed parameter settings were in accordance with our study descriptions.^28^

### SNP selection and genotyping

We searched the GeneCards database, Pubmed, HMDB, and our previous paper about metabolism changes in MASLD to identify altered changes of the phospholipid metabolism-related genes, altered phospholipids and related genes, phospholipid-related metabolites in MASLD and altered phospholipids. Also, we further chose the intersection set of genes and phospholipid-related metabolites and conducted the MASLD (NAFLD) genome-wide association (GWAS) summary data analysis to include potential causal evidence of the significant phospholipids species GWAS database through Mendelian randomization (MR) analysis. Therefore, we have the candidate genes and SNPs. We focused on the SNPs with the prevalence of variants < 10% in Han Chinese, located in the functional domain of proteins, and performed the pilot study test to identify the final 10 SNPs in the present study (Supplemental Figure S1, http://links.lww.com/HC9/A830).

**FIGURE 1:**
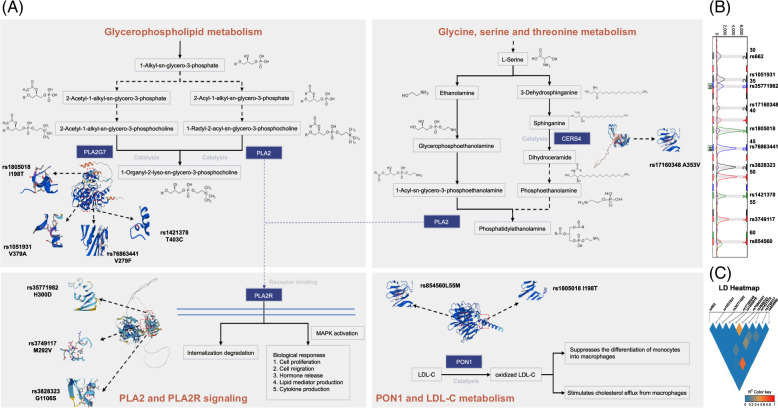
(A) The metabolic pathways of 4 phospholipid metabolism-related genes, involving glycerophospholipid, glycine, serine, threonine metabolism, PLA2 and PLA2R signalling, PONA1, and LDL-C metabolism. (B) The representative result of 10 SNPs using the SNaPshot method. (C) Linkage disequilibrium plot based on the genotype data of the PLA2G7 rs1805018, rs76863441, rs1421378, and rs1051931, PLA2R1 rs35771982, rs3828323, and rs3749117, PON1 rs662, rs854560, and CERS4 rs17160348 SNPs. Abbreviations: PLA2G7, phospholipase A2 group VII; PLA2R1, phospholipase A2 receptor 1; PON1, paraoxonase-1; CERS4, ceramide synthase 4; LDL-C, low-density lipoprotein- cholesterol; LD, linkage disequilibrium.

EDTA peripheral blood samples (2 mL per participant) were collected. Ten tagged SNPs of 4 phospholipid metabolism-related genes (phospholipase A2 group VII rs1805018, rs76863441, rs1421378, and rs1051931; PLA2R1 rs35771982, rs3828323, and rs3749117; PON1 rs662 and rs854560; and CERS4 rs17160348) were genotyped using the multiplex SNaPshot system (ABI 3730X, Applied Biosystems, Foster City, USA). The metabolic functions of these 6 genes are presented in Figure 1. The forward (F), reverse (R), and extension primers used for the PCR and extension reactions are presented in Supplemental Table S1, http://links.lww.com/HC9/A830.

The PCR was in a total reaction volume of 10 μl, which contains 1 μl template DNA (20 ng/uL), 5 μl of 2 × Taq PCR Master Mix, 1 μl of 10 μmol/ml of each primer, and 3 μl ddH_2_O. PCR reactions were performed using a PCR amplifier (Veriti 384 well, AppliedBiosystem, Foster City, USA). The detailed settings and the protocol of SNaPshot can be found in the Supplemental material, http://links.lww.com/HC9/A830.

To further confirm the accuracy of SNaPshot genotyping, a total of 30 samples were randomly selected and validated by Sanger sequencing (Shanghai Generay Biotech Co., Ltd, China). (Supplemental Figure S2, http://links.lww.com/HC9/A830).

### MR analysis

Gene-exposure data were derived from a recently published GWAS for lipoprotein-associated phospholipase A2 and coronary artery disease and myocardial infarction. We chose 7 Lp-PLA2 activity genetic variants. The GWAS data for fatty liver was obtained from the IEU OpenGWAS project. Evidence of heterogeneity of estimates was calculated from a Cochran Q test, and a significance threshold for the evidence of heterogeneity was set at *p* < 0.05. In the sensitivity analysis, we used alternate MR methods, including penalized inverse-variance weighted, weighted median and MR-Egger. The penalized inverse-variance weighted can offer robust estimates by means of attenuating partial instrumental variants. When at least 50% instrumental variables are valid and at least 50% of the weight comes from valid instrumental variables, a weighted median can offer protection against invalid instruments. The MR-Egger can provide unbiased estimates of causal effects even when all SNPs in an instrument are invalid through potential violation of the standard instrumental variable assumptions because of pleiotropy. All the analysis were performed with the packages named TwoSampleMR, MRInstruments and ieugwasr in the R software (version 4.1.0; R Development Core Team) (Supplemental Material, http://links.lww.com/HC9/A830).

### Sample size calculation

The mutation rate of PLA2R1 rs35771982 C allele was 0.40 in Asian individuals, with the settings calculated with Z-test (Unpooled) by the PASS software (NCSS, Kaysville, USA), the estimated sample size needed to be ranged from 103 to 183, to achieve a power of 80% with an alpha of 0.05 and equal allocation. Therefore, we set 200 subjects for each group when designing the research. We consecutively enrolled the patients from January 2020 to October 2022 and included patients in equal proportions for the 3 groups. If the number of people in any group has reached 200, we would stop the enrollment for this group.

### Statistical analysis

All statistical calculations were performed with SPSS software (version 25.0, IBM, Chicago, USA). The results are expressed as mean±SD. Values that not follow normally distributed were expressed as the median with IQRs. One-way ANOVA and the Kruskal-Wallis test were used to compare continuous variables. Percentages were calculated for categorical variables and compared using chi-squared test. Factors associated with moderate-severe MASLD and carotid atherosclerosis among patients, regardless of the presence of obesity, were explored through logistic regression analysis. A two-tailed *p* value of < 0.05 was considered statistically significant.

## RESULTS

### Clinical characteristics

**FIGURE 2:**
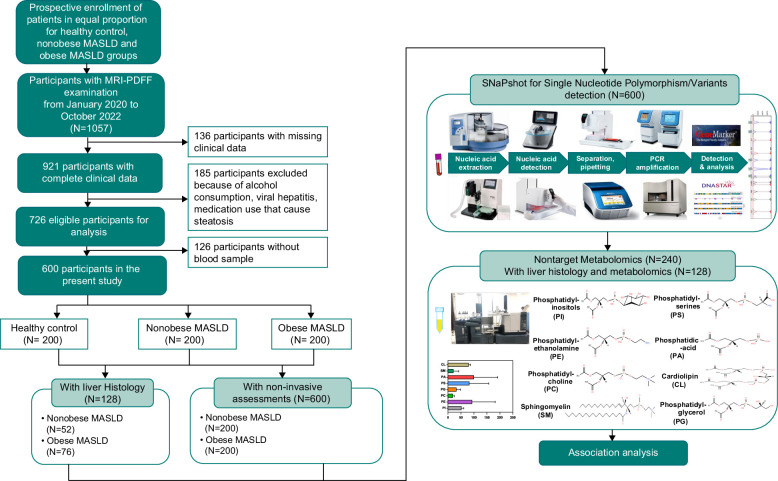
The flow chart of the present study.

A flow chart of the patients in this study is shown in Figure 2. Overall, 600 consecutive patients were recruited, including 200 healthy controls, 200 patients who are not obese with MASLD, and 200 patients with obesity. Differences in age and sex proportions were not pronounced among the 3 groups (Table 1). Patients with obesity had the highest BMI, waist circumference (WC), blood pressure, and liver enzyme levels (ALT and AST), followed by the nonobese and healthy control groups. The CHOL, TG, and LDL-C levels in patients with MASLD were higher than those in healthy controls; however, no statistical difference was found between patients with or without obesity. The nonobese and obese groups had comparable FBG levels, higher than those of the healthy controls. Patients with obesity had the highest levels of FINS, HOMA-IR, and UA and the highest proportion of patients with IR. Similarly, the LFC of the obese MASLD was 14.5(10.6–22.5)%, followed by the nonobese group (12.5 [7.1–20.2]%) and the healthy control group (4.1 [3.1–4.3]%). However, there was no difference in liver stiffness among the 3 groups (Table 1). Overall, 52 patients without obesity and 76 patients with obesity received liver biopsy and pathological assessment. Compared with the obese group, patients without obesity had a lower lobular inflammation (84.6% vs. 93.4%, *p* = 0.001) and fibrosis proportion (23.1% vs. 63.2%, *p* < 0.001); however, there was no statistical difference between steatosis and ballooning. The nonobese group had lower NAS scores(3 [2–4] vs. 3 [2–4], *p* = 0.012), steatosis, activity and fibrosis scores (3 [2–4] vs. 4 [2–5], *p* = 0.002), and proportion of steatohepatitis (23.1% vs. 51.3%, *p* = 0.001) (Supplemental Table S2, http://links.lww.com/HC9/A830). No significant difference was found between patients regardless of the presences of obesity with and without biopsy (Supplemental Table S2, http://links.lww.com/HC9/A830). In addition, 240 metabolomics individuals (80 in each group) were included. BMI, WC, and LFC were the lowest in the normal control group and highest in the obese MASLD group. Furthermore, patients without obesity with MASLD had lower levels of FINS, IR, and UA than patients with obesity (Supplemental Table S3, http://links.lww.com/HC9/A830).

**TABLE 1:** Anthropometrical and metabolic characteristics of normal controls, nonobese and obese MASLD patients

	Normal controls	Nonobese MASLD	Obese MASLD	
Characteristics	(n = 200)	(n = 200)	(n = 200)	*P*
Age, y	39.6±10.0	40.7±13.1	41.0±12.8	0.63
Male, n (%)	165 (72.5)	221 (69.5)	521 (73.0)	0.68
BMI, kg/m^2^	21.8 ± 3.0	23.4 ± 1.8	29.3 ± 3.6	<0.001^a^ ^b^ ^c^
Waist circumstance, cm	77.3 ± 7.3	83.5 ± 5.9	94.6 ± 7.9	<0.001^a^ ^b^ ^c^
Systolic blood pressure, mm Hg	119 ± 15	129 ± 20	131 ± 16	<0.001^a^ ^b^
Diastolic blood pressure, mm Hg	75 ± 11	83 ± 14	87 ± 13	<0.001^a^ ^b^ ^c^
Alanine aminotransferase, U/L†	19 (15–25)	39 (24–64)	47 (28–82)	<0.001^a^ ^b^
Aspartate aminotransferase, U/L†	24 (22–28)	31 (24–42)	34 (24–48)	<0.001^a^ ^b^
Total cholesterol, mmol/L	4.7 ± 0.9	5.3 ± 1.2	5.4 ± 1.1	<0.001^a^ ^b^
Triglycerides, mmol/L	1.1 ± 0.9	2.0 ± 1.5	2.1 ± 1.7	<0.001^a^ ^b^
HDL- cholesterol, mmol/L	1.4 ± 0.4	1.3 ± 0.6	1.2 ± 0.7	0.06
LDL- cholesterol, mmol/L	2.7 ± 0.6	3.3 ± 0.8	3.4 ± 0.8	<0.001^a^ ^b^
Fasting blood glucose, mmol/L	4.7 ± 0.5	5.2 ± 1.0	5.2 ± 0.9	<0.001^a^ ^b^
Fasting insulin, μU/mL†	4.8 (3.4–6.6)	8.5 (6.2–11.8)	10.6 (8.0–17.5)	<0.001^a^ ^b^ ^c^
HOMA-IR†	1.0 (0.7–1.3)	2.0 (1.3–2.8)	2.3 (1.7–3.8)	<0.001^a^ ^b^ ^c^
HOMA-IR > 2.5, n (%)	5 (2.5%)	60 (30.0%)	88 (44.0%)	<0.001^a^ ^b^ ^c^
Uric acid, μmol/L	320 ± 90	382 ± 109	423 ± 106	<0.001^a^ ^b^ ^c^
Liver fat content, %†	4.1 (3.1–4.3)	12.5 (7.1–20.2)	14.5 (10.6–22.5)	<0.001^a^ ^b^ ^c^
Liver stiffness, kPa	4.9 ± 1.8	5.4 ± 2.1	5.9 ± 2.2	0.15

† Continuous variables are expressed as median with IQR for non-Gaussian distribution.

a *P*<0.05 between normal controls and nonobese MASLD.

b *P*<0.05 between normal controls and obese MASLD.

c *P*<0.05 between nonobese MASLD and obese MASLD.

Abbreviations: HDL-cholesterol, high-density lipoprotein-cholesterol; HOMA-IR, Homeostasis Model Assessment of Insulin Resistance; LDL-cholesterol, low-density lipoprotein-cholesterol.

### Mendelian randomization

Cochran Q test showed that there was heterogeneity among genetic variants for MASLD (*p* = 0.72) and MI (*p* = 0.91). The *p* values of Cochran Q-test and MR-Egger intercept were 0.71 and 0.91, respectively, indicating no evidence of potential horizontal pleiotropy and heterogeneity. In sensitivity analyses, the inverse-variance weighted method (*p* = 0.07) and the weighted median method (*p* = 0.11) showed that Lp-PLA2 activity might have a broadline effect on the risk of MASLD in Europeans. (Supplemental Material, http://links.lww.com/HC9/A830).

### Genotypes of genes involved in phospholipid metabolism and MASLD

Ten SNPs of 4 phospholipid metabolism-related genes were selected, and their distributions in all 3 groups followed Hardy-Weinberg equilibrium. A representative SNaPshot result is shown in Figure 1. The evaluated SNPs exhibited low linkage disequilibrium, with the highest observed between PLA2R1 rs35771982 and PLA2R1 rs3749117 (Figure 1). Alleles of 4 Single nucleotide polymorphism loci were distributed differently among the 3 groups: PON1 rs854560, PLA2R1 rs35771982, rs3749117, and CERS4 rs17160348. The proportion of the PON1 rs854560 adenine pairs (AA) genotype was significantly higher in patients with obesity with MASLD than in healthy controls (93.0% vs. 87.0%, *p* = 0.043). Patients without obesity with MASLD had the highest proportion of the PLA2R1 rs35771982 Guanine, G (GG) genotype, followed by patients with obesity with MASLD and healthy controls (61.5% vs. 45.5% vs. 49.0%, *p* = 0.028). The frequency of the Thymine, T (TT) genotype of PLA2R1 rs3749117 in obese patients with MASLD was significantly higher than that in the healthy controls and patients without obesity with MASLD (61.5% vs. 48.5% vs. 45.5%, *p* = 0.025), and there was no significant difference between the healthy controls and patients without obesity with MASLD. The distribution of CERS4 rs17160348 Cytosine-Thymine (CT)/TT in the healthy control group was the lowest among the 3 groups (17.0%, 22.5%, and 28.5%, *p* = 0.014) (Table 2).

**TABLE 2:** Distribution of polymorphism among normal controls, nonobese, and obese patients with MASLD

Gene SNP	Normal controls (n = 200)	Nonobese MASLD (n = 200)	Obese MASLD (n = 200)	*p*
PLA2G7 rs1805018	—	—	—	0.13
AA, n (%)	153 (76.5)	170 (85.0)	172 (86.0)	—
AG, n (%)	45 (22.5)	29 (14.5)	27 (13.5)	—
GG, n (%)	2 (1.0)	1 (0.5)	1 (0.5)	—
PLA2G7 rs76863441	—	—	—	0.38
CC, n (%)	179 (89.5)	184 (92.0)	182 (91.0)	—
CG, n (%)	21 (10.5)	15 (7.5)	18 (9.0)	—
AA, n (%)	0 (0)	1 (0.5)	0 (0)	—
PLA2G7 rs1421378	—	—	—	0.11
AA, n (%)	110 (55.0)	123 (61.5)	119 (59.5)	—
AG, n (%)	79 (39.5)	73 (36.5)	66 (33.0)	—
GG, n (%)	11 (5.5)	4 (2.0)	15 (7.5)	—
PLA2G7 rs1051931	—	—	—	0.20
GG, n (%)	157 (78.5)	150 (75.0)	158 (79.0)	—
GA, n (%)	38 (19.0)	49 (24.5)	36 (18.0)	—
AA, n (%)	5 (2.5)	1 (0.5)	6 (3.0)	—
PON1 rs662	—	—	—	0.42
CC, n (%)	87 (43.5)	80 (40.0)	96 (48.0)	—
CT, n (%)	94 (47.0)	97 (48.5)	89 (44.5)	—
TT, n (%)	19 (9.5)	23 (11.5)	15 (7.5)	—
PON1 rs854560	—	—	—	**0.038** ^a^
AA, n (%)	174 (87.0)	184 (92.0)	186 (93.0)	
AT, n (%)	16 (8.0)	14 (7.0)	12 (6.0)	—
TT, n (%)	10 (5.0)	2 (1.0)	2 (1.5)	—
PLA2R1 rs35771982	—	—	—	**0.011** ^b^,^c^
GG, n (%)	98 (49.0)	123 (61.5)	91 (45.5)	—
GC, n (%)	87 (43.5)	64 (32.0)	86 (43.0)	—
CC, n (%)	15 (7.5)	13 (6.5)	23 (11.5)	—
PLA2R1 rs3828323	—	—	—	0.29
CC, n (%)	114 (57.0)	115 (57.5)	121 (60.5)	—
CT, n (%)	69 (34.5)	66 (33.0)	71 (35.5)	—
TT, n (%)	17 (8.5)	19 (9.5)	8 (4.0)	—
PLA2R1 rs3749117	—	—	—	**0.01** ^a^,^c^
TT, n (%)	97 (48.5)	91 (45.5)	123(61.5)	—
TC, n (%)	88 (44.0)	86 (43.0)	64 (32.0)	—
CC, n (%)	15 (7.5)	23 (11.5)	13 (6.5)	—
CERS4 rs17160348	—	—	—	**0.012** ^b^,^a^
CC, n (%)	166 (83.0)	147 (77.5)	143 (71.5)	—
CT, n (%)	31 (15.5)	40 (16.5)	40 (20.0)	—
TT, n (%)	3 (1.5)	13 (6.0)	17 (8.5)	—

a *p* < 0.05 between normal controls and obese MASLD.

b *p* < 0.05 between normal controls and nonobese MASLD.

c *p* < 0.05 between nonobese MASLD and obese MASLD.

Abbreviations: AA, adenine pairs; AG, Adenine, G; AT, Adenine, T; CC, Cytosine, C; CERS4, ceramide synthase 4; CT, Cytosine, T; GA, Guanine, A; GC, Guanine, C; GG, Guanine, G; MASLD, Metabolic dysfunction–associated steatotic liver disease; PLA2G7, phospholipase A2 group VII; PLA2R1, phospholipase A2 receptor 1; PON1, paraoxonase-1; TT, Thymine, T.

### Clinical characteristics of different genotypes

We further compared the clinical characteristics of the groups stratified according to the genotypes of the significant genes. For the PON1 rs854560 AA, Adenine, T (AT), and TT genotypes, there were 544, 42, and 14 individuals, respectively. The TT group had the lowest BMI and diastolic blood pressure (TT vs. AT vs. AA: 21.8±3.8 vs. 24.5±3.6 vs. 24.9±3.96 kg/m^2^, *p* = 0.014; 75.2±10.6 vs. 82.6±12.8 vs. 78.3±16.3 mm Hg, *p* = 0.025). Additionally, FINS and LFC of the TT group were also significantly lower than those of the other 2 groups (TT vs. AT vs. AA:4.0 [2.9–5.3] vs. 7.2 [5.2–10.8]) vs. 8.3 (5.1–11.8) μU/mL, *p* = 0.008; 8.2 (4.5–15.3)% vs. 10.7 (7.2–18.6)% vs. 15.9 (8.7–24.5)%, *p* < 0.001]. There were no significant differences in age, gender, waist circumference, systolic blood pressure, liver enzymes, blood lipids, fasting blood glucose, HOMA-IR, UA, or liver hardness between the three groups (all *p* > 0.05, Supplemental Table S4, http://links.lww.com/HC9/A830).

Furthermore, for PLA2R rs35771982 genotype, 312, 237, and 51 individuals had the GG, GC, and Cytosine, C (CC) genotypes, respectively. As shown in Table 4, only diastolic blood pressure was statistically different among the 3 groups (CC vs. CG vs. GG:81.3±12.7 vs. 82.0±13.8 vs. 87.2±11.2 mm Hg, *p* = 0.025). There were no differences in liver enzyme, blood lipids, blood glucose, UA, LFC, or liver stiffness among the 3 groups (Supplemental Table S5, http://links.lww.com/HC9/A830).

The group of TT in PLA2R1 rs3749117 exhibited the lowest levels of BMI (24.3±3.7 vs. 24.4±3.4 vs.25.2±3.9 kg/m^2^, *p* = 0.024) and WC (84.4±10.6 vs. 85.1±8.5 vs. 88.4±9.0 cm, *p* = 0.001). No significant differences were found among the 3 groups regarding age, gender, blood pressure, liver enzymology, metabolic indicators (blood lipids, blood glucose, and UA), LFC, and liver stiffness (Supplemental Table 6, http://links.lww.com/HC9/A830).

The carriers of the CERS4 rs17160348 T-allele had a large waist circumference (85.7±11.0 vs. 86.4±9.2 vs. 92.0±11.9 cm, *p* = 0.033). CHOL and LDL-C were significantly lower in the CC group than in the CT and TT groups (Supplemental Table 7, http://links.lww.com/HC9/A830).

### Effects of gene polymorphisms on disease severity

**FIGURE 3:**
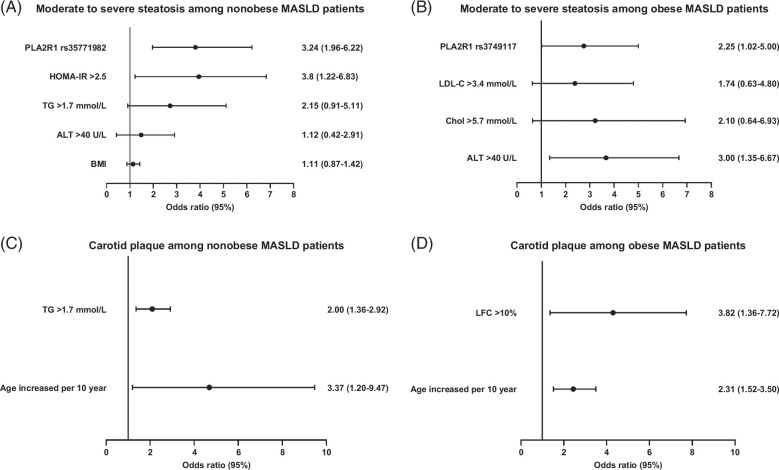
Multivariate logistic regression analysis of factors associated with moderate-to-severe steatosis (A, B) and carotid plaque (C, D).

The univariate analysis revealed that BMI, ALT > 40 U/L, TG > 1.7 mmol/L, IR, and PLA2R1 rs35771982 were significantly associated with an increased risk of moderate-severe MASLD in patients without obesity. In obese individuals, ALT > 40 U/L, CHOL > 5.7 mmol/L, LDL-C > 3.4 mmol/L, and PLA2R1 rs3749117 were identified as risk factors for moderate-severe MASLD (Table 3). After multivariate adjustments, IR (OR: 3.80, 95% CI: 1.22–6.83, *p* = 0.021) and PLA2R1 rs35771982 (OR: 3.24, 95% CI: 1.96–6.22, *p* = 0.038) remained significantly associated with nonobese moderate-severe steatosis. ALT > 40 U/L (OR: 3.00, 95% CI:1.35–6.67, *p* = 0.007) and PLA2R1 rs3749117 were independent predictors of moderate-severe steatosis (Figure 3).

**TABLE 3:** Factors associated with moderate-to-severe MASLD and carotid atherosclerosis among nonobese and obese MASLD

	Moderate-to-severe MASLD				Carotid atherosclerosis			
	Nonobese		Obese		Nonobese		Obese	
Factors	OR (95% CI)	*p*	OR (95% CI)	*p*	OR (95% CI)	*p*	OR (95% CI)	*p*
Age increased per 10 y	1.15 (0.96–1.39)	0.14	1.76 (0.58–5.33)	0.32	2.72 (1.09–6.77)	0.032	5.29 (2.64–10.61)	0.001
Male	1.73 (0.83–3.62)	0.15	0.87 (0.38–2.00)	0.74	2.53 (0.76–8.34)	0.13	0.97 (0.32–2.94)	0.95
BMI (kg/m^2^)	1.23 (1.01–1.50)	0.047	1.15 (0.99–1.36)	0.07	1.26 (0.87–1.84)	0.53	0.92 (0.76–1.11)	0.37
Increased WC	1.02 (0.96–1.08)	0.56	1.05 (0.99–1.11)	0.13	1.05 (0.96–1.15)	0.30	1.03 (0.96–1.10)	0.40
ALT > 40 U/L	2.82 (1.39–5.75)	0.004	4.28 (2.01–9.07)	0.017	2.27 (0.74–6.92)	0.15	1.12 (0.87–1.64)	0.29
CHOL > 5.7 mmol/L	1.04 (0.47–2.30)	0.83	3.42 (1.33–8.78)	0.011	0.48 (0.13–1.73)	0.26	0.67 (0.24–1.87)	0.84
TG >1 .7 mmol/L	2.65 (1.33–5.30)	0.006	1.83 (0.64–5.23)	0.26	1.89 (1.31–2.73)	0.001	2.07 (0.82–5.22)	0.12
LDL-C >3.4 mmol/L	1.04 (0.53–2.06)	0.90	3.05 (1.37–6.72)	0.006	0.47 (0.16–1.40)	0.17	1.58 (0.63–3.92)	0.33
HOMA-IR >2.5	4.62 (1.88–11.32)	0.001	1.78 (0.82–3.90)	0.15	0.94 (0.28–3.17)	0.92	1.23 (0.45–3.32)	0.69
Hyperuricemia	1.32(0.68–2.58)	0.41	1.73 (0.72–4.22)	0.23	1.11 (0.39–3.12)	0.84	0.77 (0.31–1.91)	0.57
LFC >10%	—	—	—	—	1.08 (0.97–1.15)	0.17	2.82 (1.13–6.05)	0.026
Liver stiffness > 6.1kPa	1.25 (0.72–2.01)	0.18	0.66 (0.28–1.56)	0.34	1.27 (0.81–1.76)	0.22	1.58 (0.84–2.23)	0.22
PON1 rs854560	0.66 (0.16–2.67)	0.56	0.50 (0.24–0.89)	0.03	0.99 (0.98–1.01)	0.18	0.99 (0.98–1.02)	0.41
PLA2R1 rs35771982	2.43 (1.24–4.78)	0.01	0.90 (0.44–1.84)	0.78	1.10 (0.37–3.29)	0.87	0.60 (0.07–5.24)	0.64
PLA2R1 rs3749117	1.15 (0.58–2.27)	0.70	2.33 (1.12–4.81)	0.025	0.83 (0.09–8.62)	0.88	1.41 (0.45–4.43)	0.56
CERS4 rs17160348	0.70 (0.31–1.58)	0.39	1.04 (0.43–2.55)	0.93	0.70 (0.21–2.36)	0.57	1.46 (0.58–3.70)	0.42

Abbreviations: CERS4, ceramide synthase 4; CHOL, total cholesterol; HOMA-IR, Homeostasis Model Assessment of Insulin Resistance; LDL-C, low-density lipoprotein-cholesterol; LFC, liver fat content; PLA2G7, phospholipase A2 group VII; PLA2R1, phospholipase A2 receptor 1; PON1, paraoxonase-1; TG, triglycerides; WC, waist circumstance.

Age was found to be a common risk factor for carotid plaque in both nonobese (OR: 3.37, 95% CI: 1.20–9.47, *p* = 0.021) and patients with obesity (OR: 2.31, 95% CI:1.52–3.50, *p*<0.001). Additionally, TG > 1.7 mmol/L (OR: 2.00, 95% CI:1.36–2.92, *p* < 0.001) was significantly associated with a carotid plaque in patients without obesity and LFC > 10% (OR: 3.82, 95% CI:1.36–7.72, *p* < 0.011) was identified as an independent predictor of carotid plaque in patients with obesity (Table 3, Figure 3).

Overall, 76 patients with obesity and 52 patients without obesity underwent liver histology examination. The univariate and multivariate logistic regression analyses were repeated and confirmed that GC/CC in PLA2R1 rs35771982 was the only risk factor associated with steatohepatitis in patients without obesity (OR: 2.61, 95% CI:1.15–3.87, *p* = 0.028, Supplemental Table 8, http://links.lww.com/HC9/A830), whereas TC/CC in PLA2R1 rs3749117 was significant factor associated with steatohepatitis in patients with obesity (OR:1.32,95% CI:1.12–1.83, *p* = 0.019, Supplemental Table 9, http://links.lww.com/HC9/A830).

### Gene polymorphism and altered metabonomics

**FIGURE 4:**
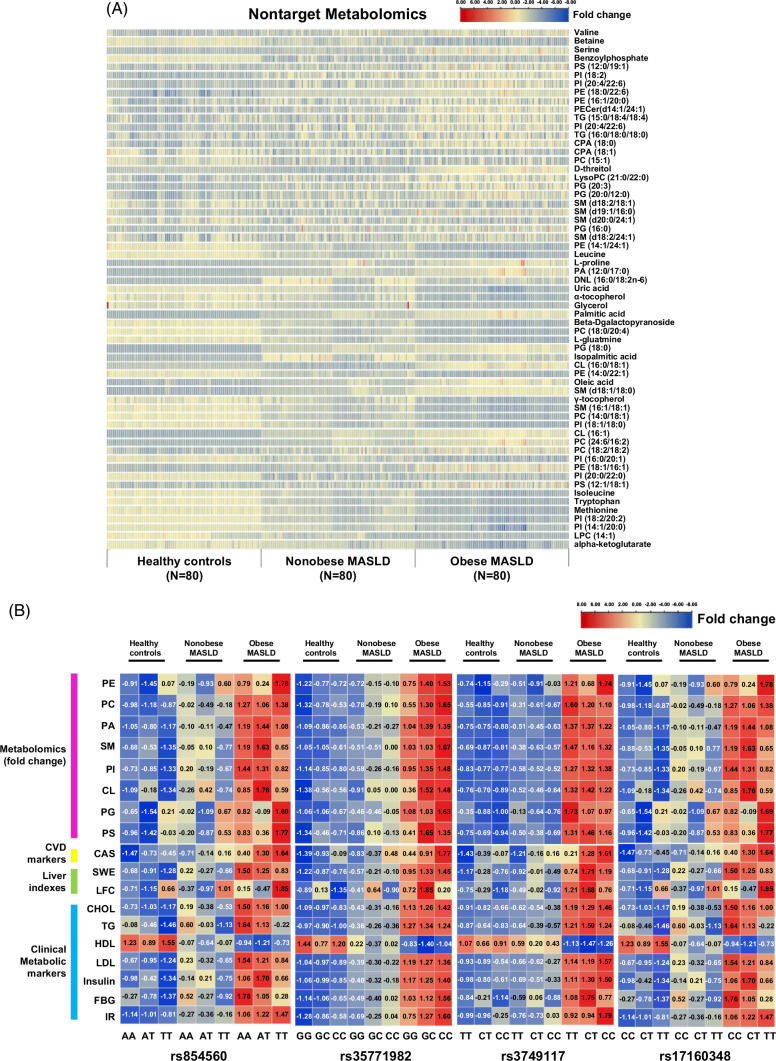
Gene polymorphisms and serum metabolite detected through metabonomics. (A) The heatmap of metabolites concentrations among healthy controls, nonobese patients with MASLD, and obese patients. (B) Comparison of different lipid metabolites concentrations, cardiovascular disease (CVD) markers, liver indexes, and clinical metabolic markers among different genotypes. Abbreviations: AA, adenine pairs; AT, Adenine, T; CC, Cytosine, C; CT, Cytosine, T; GC, Guanine, C; GG, Guanine, G; PS, phosphatidyl serine; TT, Thymine, T; TPI, thymine phosphatidyl inositol; PE, phosphatidyl ethanolamine; TG, triglyceride; CPA, cyclic phosphatidic acid; PC, phosphatidylcholine; PG, phosphoglyceride; SM, sphingomyelin; PA, phosphatidic acid; DNL, de novo lipogenesis; CL, cardiolipin; CAS, carotid atherosclerosis; CVD, cardiovascular disease; SWE, shear wave elastography; LFC, liver fat content; CHOL, cholesterol; TG, triglyceride; HDL-C, high-density lipoprotein- cholesterol; LDL-C, low density lipoprotein- cholesterol; FBG, fasting blood glucose; FINS, fasting insulin; HOMA-IR, Homeostatic Model Assessment of Insulin Resistance.

We conducted serum nontarget metabolomics detection in 240 individuals. The main differential metabolites among normal controls, patients without obesity with MASLD, and patients with obesity with MASLD were phospholipids, amino acids, UA, alpha-ketoglutarate, and tocopherol (Figure 4A). We investigated the associations between phospholipid metabolism-related gene polymorphisms and phospholipids as well as the CVD markers, liver indexes, and clinical metabolic markers among the normal control, nonobese MASLD, and obese MASLD groups, respectively (Figure 4B).

**FIGURE 5:**
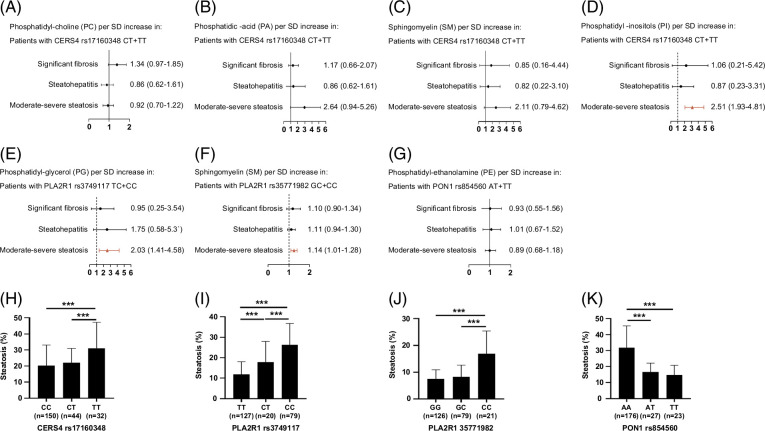
Association analysis between serum metabolites level and liver histology features according to the gene polymorphisms (A-G) and steatosis comparison among groups based on different genotypes. (A-D) CERS4 rs17160348; (E) PLA2R1 rs3749117; (F) PLA2R1 rs35771982; (G) PON1 rs854560. (H) CERS4 rs17160348; (I) PLA2R1 rs3749117; (J) PLA2R1 rs3749117; and (K) PON1 rs854560. Abbreviations: AA, adenine pairs; AT, Adenine, T; CC, Cytosine, C; CT, Cytosine, T; GC, Guanine, C; GG, Guanine, G; PLA2R1, phospholipase A2 receptor 1; PON1, paraoxonase-1; CERS4, ceramide synthase 4; PS, phosphatidyl serine; PI, phosphatidyl inositol; PE, phosphatidyl ethanolamine; PC, phosphatidylcholine; PG, phosphoglyceride; PA, phosphatidic acid; SM, sphingomyelin; TT, Thymine, T.

We further conducted the association analysis to explore the association between metabolites and histological features after stratification for genotypes. The results showed that after stratification for the rs17160348 genotypes, the association between PI and moderate-severe steatosis was evident in patients who were carrying the T-allele (OR: 2.51; 95% CI: 1.93–4.81). Furthermore, the association between phosphoglyceride and moderate-severe steatosis was obvious in patients with the CT or CC genotype (OR: 2.03; 95% CI: 1.41–4.58) and CG or CC genotype (OR: 1.14; 95% CI: 1.01–1.28) when stratifying for the rs3749117 and rs37551982 genotypes, respectively (Figure 5A-G). For liver steatosis, the CERS4 rs17160348 TT genotype, PLA2R1 rs3749117 CC genotype, and PLA2R1 rs3749117 CC genotype had significantly higher steatosis proportions than the other 2 genotypes, respectively, while PON1 rs854560 TT genotype had the lower steatosis proportion than the wild genotype (Figure 5).

## DISCUSSIONS

Our major findings indicate that PLA2R1 rs35771982 and CERS4 rs17160348 may increase susceptibility to MASLD in nonobese patients, while PON1 rs854560, PLA2R1 rs3749117, and CERS4 rs17160348 may confirm susceptibility to MASLD in obese patients. Further analysis showed that carriers of the T-allele of CERS4 rs17160348 were strongly linked to higher serum CHOL and LDL-C levels than that in wild-type (CC), while carriers of the T-allele of PON1 rs854560, which displayed significantly lower LFC levels than wild-type (AA) carriers. The presence of moderate-to-severe steatosis in nonobese MASLD may be associated with the effects of PLA2R1 rs35771982, whereas rs3749117 mutations may have pronounced effects on moderate-to-severe steatosis in obese MASLD patients.

PON1, a member of the paraoxon family, is a calcium-dependent protease that is mainly expressed in the liver and secreted into the blood along with HDL-C. Its active components can bind to HDL-C and protect against the excessive accumulation of oxidation products in LDL-C, ultimately inhibiting atherosclerosis formation.^29^ PON1 exerts a protective effect against oxidative stress and inflammation and may play a beneficial role in MASLD. Furthermore, PON1 deficiency is associated with oxidative stress and metabolic alterations, leading to liver steatosis in mice fed with a high-fat, high-cholesterol diet.^29^ The common polymorphism in the coding region of PON1, that is, leucine replaces methionine(L55M) at the rs854560 locus, affects PON1 concentration. The PON1 L allele presented with more mRNA than the PON1 M allele, leading to an increase in PON1 levels. In addition, the glutamine-substituted arginine at position 192 (Q192R) is related to PON1 activity because it can lead to different catalytic activities for synthetic substrates.^30^ A study in Romania included 81 patients with MASLD with elevated transaminase levels diagnosed by abdominal ultrasound and 81 healthy controls and demonstrated that patients with MASLD had lower serum PON1 levels and that the rs854560 mutation was a risk factor for MASLD (OR:3.44, *p* = 0.04).^31^ Studies have shown that the PON1 rs854560 polymorphism affects the efficacy of PON1 in inhibiting LDL oxidation.^31^ However, in the present study, patients with the T-allele had lower LFC, BMI, and FINS levels. In patients with metabolomics, the TT genotype group had the highest level of phosphatidylethanolamine but the lowest levels of SM, LFC, and FBG. The present results show that the rs854560 mutation seems to be a protective factor against the presence of MASLD. This may be because the sample size included in this study was larger, and we focused on the Chinese Han population, utilizing the precise technique of MRI-proton density fat fraction to quantify liver steatosis.

PLA2R1 belongs to the mannose receptor family and is an important type I transmembrane protein involved in regulating phospholipid metabolism. PLA2R1 binds to ligand-secreted phospholipase A2 (sPLA2). This process inhibited the sPLA2’s activity and formed complexes that can be internalized into cells for degradation to negatively regulate the key enzyme sPLA2 in phospholipid metabolism.^32^ This procedure can also activate the MAPK and JAK pathways to trigger the downstream transcription and expression of target genes, thereby mediating the regulation of various biological effects of sPLA2 on cells.^33^ Studies reported that the activation of PLA2R1 affects cell proliferation, migration, reactive oxygen species-mediated aging, and inflammatory mediator release.^33^ PLA2R1 has been reported in a series of studies on membranous nephropathy and is widely used in clinical practice. A meta-analysis showed that the sensitivity and specificity of serum anti-PLA2R1 in diagnosing idiopathic membranous nephropathy were 68% and 97%, respectively.^34^ In addition, many SNPs have been reported to be a risk factor for idiopathic membranous nephropathy(rs4664308, rs3749117, rs35771982, and rs3823323).^35^,^36^,^37^ In a study from Japan involving 941 consecutive inpatients presenting chest pain, it was found that PLA2R1 rs3749117 mutation was a risk factor for increased carotid intima-media thickness measured by a high-resolution B-mode ultrasonography (OR = 1.93, 95% CI:1.17–3.19, *p* = 0.01).^16^ However, no relevant studies have explored the role and potential mechanism of action of PLA2R1 in the pathogenesis of MASLD. This study found, that PLA2R1 rs35771982 and rs3749117 were associated with susceptibility to MASLD with and without obesity, as well as progression to steatohepatitis. In addition, patients with the rs3749117 CC genotype had the lowest phosphoglyceride concentrations, which was found to be a protective factor against liver steatosis in our previous study. Therefore, we found that the rs3749117 mutation might be a risk factor for MASLD by reducing phosphoglyceride concentration in blood circulation. Molecular experiments are still needed for further verification of these findings.

CERS4 is an enzyme located in the mitochondrial membrane that participates in the ceramide metabolic pathway. Its major function is to facilitate the acylation of ceramides into sphingosines and fatty acids.^38^ Ceramide is a small component of the lipid layer in many tissues. However, the accumulation of a small amount of ceramide may cause a variety of lipid metabolism-related diseases, including MASLD and diabetes. The accumulation of ceramide and sphingomyelin in the livers of mice fed a high-fat diet for 3 weeks could eventually lead to fatty liver and could be reversed by targeting dihydroceramide desaturase.^39^ Changes in the biological characteristics of ceramides and dihydroceramides through the targeted knockout of dihydroceramide desaturase can improve insulin resistance and hepatocyte steatosis in mouse models.^40^ Our present study reports that CERS4 rs17160348 is associated with obese MASLD and that patients with the T allele have greater WC and higher CHOL and LDL-C levels. Whether the CERS4 rs17160348 mutation leads to a decrease in its enzyme activity and the accumulation of ceramide in vivo remains to be elucidated.

A limitation of the study was that only a minority of our study group (128 of 400 patients with MASLD) underwent liver biopsy, and 240 individuals underwent lipidomic analysis. Therefore, a larger sample size is required for clinical research is still needed to verify these findings. Furthermore, we did not use immunohistochemistry and western blotting to detect the expression of liver-related genes, nor did we conduct metabolomics of the liver tissue. Finally, we did not explore or validate the relationship between phospholipid metabolism-related genotypes and metabolite changes.

Mutations in PLA2R1 rs35771982 and CERS4 rs17160348 are associated with susceptibility to MASLD in nonobese individuals, while PLA2R1 rs35771982 mutation is a risk factor for moderate-to-severe steatosis and steatohepatitis in nonobese patients. Polymorphisms in phospholipid metabolism-related genes play a crucial role in the occurrence and development of MASLD.

## Supplementary Material

SUPPLEMENTARY MATERIAL
