# Supplementary material for: Phospholipid metabolism-related genotypes of PLA2R1 and CERS4 contribute to nonobese MASLD
Source: Hepatol Commun. 2024 Jun 5;8(6):e0388. doi: 10.1097/HC9.0000000000000388 (PMC11155565; doi:10.1097/HC9.0000000000000388)
Supplement: SUPPLEMENTARY MATERIAL [file hc9-8-e0388-s001.doc]

**Supplementary Appendix**

**Phospholipid metabolism-related genotypes of PLA2R1 and CERS4 contribute to** **nonobese MASLD**

Congxiang Shao^1#^, Junzhao Ye^1#^, Zhi Dong^2^, Bing Liao^3^, Shiting Feng^2^, Hu Shixian^1,4*^, Bihui Zhong^1*^

^1^Department of Gastroenterology of the First Affiliated Hospital, Sun Yat-sen University, No. 58 Zhongshan II Road, Yuexiu District, Guangzhou, China

^2^Department of Radiology of the First Affiliated Hospital, Sun Yat-sen University, No. 58 Zhongshan II Road, Yuexiu District, Guangzhou, China

^3^Department of Pathology of the First Affiliated Hospital, Sun Yat-sen University, No. 58 Zhongshan II Road, Yuexiu District, Guangzhou, China

^4^Institute of Precision Medicine, The First Affiliated Hospital, Sun Yat-Sen University, Guangzhou, Guangdong, China.

Congxiang Shao, MD, Ph.D. ^1^, shaocx6@mail.sysu.edu.cn

Junzhao Ye, MD, Ph.D. ^1^, yejzh@mail2.sysu.edu.cn

Zhi Dong, MD, Ph.D. ^2^, dongzh7@mail.sysu.edu.cn

Bing Liao, MD, Ph.D.^3^, liaob@mail.sysu.edu.cn

Shiting Feng, MD, Ph.D. ^2^, fengsht@mail.sysu.edu.cn

Shixian Hu, Ph.D. ^1,4^, hushx9@mail.sysu.edu.cn

Bihui Zhong, MD, Ph.D. ^1^, zhongbh@mail.sysu.edu.cn

^#^Congxiang Shao and Junzhao Ye contributed equally to this paper.

* Hu Shixian and Bihui Zhong contributed equally to this paper.

***Correspondence to:**

Hu Shixian Ph.D., No. 58 Zhongshan II Road, Yuexiu District, Guangzhou, China, 510080. E-mail: hushx9@mail.sysu.edu.cn

TEL: (8620) 020-87332200; FAX: (8620) 020-87332200

ORCID: 0000-0002-1190-0325

Bihui Zhong, MD, Ph.D., No. 58 Zhongshan II Road, Yuexiu District, Guangzhou, China, 510080. E-mail: zhongbh@mail.sysu.edu.cn

TEL: (8620)87755766; FAX: (8620)87750632

ORCID: 0000-0002-8483-6854

**Supplementary material contents**

[Supplementary Instructions and Methods 5](#__RefHeading___Toc154011985)

[**SNP Genotyping** 5](#__RefHeading___Toc154011986)

[**Supplementary Tables** 6](#__RefHeading___Toc154011987)

[**Supplementary Table 1** Forward, reverse, and extension primers of the 10 tag SNPs for PCR. 6](#__RefHeading___Toc154011988)

[**Supplementary Table 2.** Anthropometrical and metabolic characteristics of nonobese and obese MASLD patients with/without liver biopsy. 7](#__RefHeading___Toc154011989)

[**Supplementary Table 3.** Anthropometrical and metabolic characteristics of nonobese and obese MASLD patients with metabonomics. 9](#__RefHeading___Toc154011990)

[**Supplementary Table 4** Characteristics of the study cohort based on the PLA2R rs35771982 genotype. 11](#__RefHeading___Toc154011991)

[**Supplementary Table 5** Characteristics of the study cohort based on the PLA2R rs35771982 genotype. 12](#__RefHeading___Toc154011992)

[**Supplementary Table 6** Characteristics of the study cohort based on the PLA2R rs3749117 genotype. 13](#__RefHeading___Toc154011993)

[**Supplementary Table 7** Characteristics of the study cohort based on the CERS4 rs17160348 genotype. 14](#__RefHeading___Toc154011994)

[**Supplementary Table 8** Factors associated with moderate-severe steatosis, steatohepatitis and significant fibrosis in nonobese MASLD patients. 15](#__RefHeading___Toc154011995)

[**Supplementary Table 9** Factors associated with moderate-severe steatosis, steatohepatitis and significant fibrosis in obese MASLD patients. 16](#__RefHeading___Toc154011996)

[**Supplementary Figure** 17](#__RefHeading___Toc154011998)

[**Supplementary Figure 1.** Flowchart for the genes/variants selection process 17](#__RefHeading___Toc154011999)

[**Supplementary Figure 2.** The sequencing validation of SNaPshot method in SNP genotyping of 10 loci 20](#__RefHeading___Toc154012000)

[**Mendelian randomization analysis** 21](#__RefHeading___Toc154012001)

Supplementary Instructions and Methods

**SNP Genotyping**

The PCR was in a total reaction volume of 10 μl, which contains 1 μl template DNA (20ng/uL), 5 μl of 2×Taq PCR Master Mix, 1μl of 10 μmol/ml of each primer and 3 μl ddH_2_O. PCR reactions were performed using a PCR amplifier (AppliedBiosystem, Foster City, USA). The detailed settings and the protocol of SNaPshot could be found in the supplement material. with the following settings: initial denaturation at 94 °C for 5 min, 35 cycles of denaturation at 94 °C for 20 s, annealing at 60 °C for 30 s and extension at 72 °C for 30 s, and a final extension step at 72 °C for 3 min. PCR products were purified with the reaction volume: 4.0 μl of PCR products, 0.15 μl of 20 μ/μl Exonuclease I (Thermo, USA), 0.5 μl of 1 μ/μl thermosensitive alkaline phosphatase (FastAP ™, Thermo, USA), 0.6 μl ExoI buffer and 0.75 μl ddH2O. PCR reactions were conducted with 37 °C for 40 min and 85 °C for 15 min. SNaPshot extension reactions were performed following the instructions of SNaPshot MuLtiple kit (Applied Biosystems, Foster City, USA) with slight revisions. The total 5 μl reaction volume included 3 μl of purified PCR products, 0.5 μl of SNaPshot Multiplex Ready Reaction Mix, 1 μl of 10 μmol/ml pooled extension primer and 0.5 μl ddH2O. Extension reactions were performed using the following procedures: pre-denaturation at 95 °C for 30 s, followed by 40 cycles of denaturation at 95 °C for 5 s, annealing at 52 °C for 5 s, extension at 60 °C for 5 s and 72 °C for 3 min. The products 2.0 μl and 8.0 μl formamide were mixed and then were denatured at 95 °C for 5 min. The fluorescently labelled fragments were separated by capillary electrophoresis on an ABI PRISM 3730 XL Genetic Analyzer (Applied Biosystems, Foster City, USA).

**Supplementary Tables**

**Supplementary Table 1 Forward, reverse, and extension primers of the 10 tag SNPs for PCR.**

| **Gene** | **SNP** | **Forward and reverse primers (5’-3’)** | **Extension primers (5’-3’)** |
| --- | --- | --- | --- |
| PLA2G7 | rs1805018  I198T | F: GAAACATGGGTCAGATGAGG  R: AGCTAGGAGCATAACTTGCC | tgactgactgactGGTAGAGCCAAGACTTGTCCCCT |
| PLA2G7 | rs7686344  V279F | F: GAGAGGTAGATGTTTTAGCC  R: ATAGGACTCTATTGATAGGG | tgactgactgactgactGACATTCTTTTGGTGGAGCAACG |
| PLA2G7 | rs1421378  T403C | F: TTCTCCTCAGACCTAAGTGC  R: TGCCGATCAGATTTACTCCC | gactgactgactgactgactgactgactTGCGCATTTTCCGCACTCCG |
| PLA2G7 | rs1051931  V379A | F: GCTTTGTCCTGAGATTCATC  R: AGACCAACAAGACCAGTACC | gactGGGAGACATAGATTCAAATG |
| PLA2R1 | rs35771982  H300D | F: ACCTTTCCCGTCATTGACTG  R: CTGTGTAAGGGTGCAAGATG | tgactgactgAGACCACTGCCAGCCAGCGT |
| PLA2R1 | rs3828323  G1106S | F: CATGGATCATAGTGACAAGG  R: TGAGGAAGGACTGGTGATAC | actgactgactgactgactGGATACATATCAGATGTATTTACAC |
| PLA2R1 | rs3749117  M292V | F: CTGTGTAAGGGTGCAAGATG  R: ACCTTTCCCGTCATTGACTG | gactgactgactgactgactgactgactgactATCCAGCTGATTGAGGCCCA |
| PON1 | rs662  Q192R | F: AAGGATTGTATCGGCAGGAC  R: ACATACGACCACGCTAAACC | CCAAATACATCTCCCAGGAT |
| PON1 | rs854560  L55M | F: TTTCTGGCAGAAACTGGCTC  R: TGGTTCAATGTAGACCGAAG | gactgactgactgactgactgactgactgactgactCAGAAACTGGCTCTGAAGAC |
| CERS4 | rs17160348  A353V | F: TCTTTAGCTGCAGAGGTTCC  R: GGCTACTACTTCTTCAACGG | actgactgactAATCAGACTCCAGTGAGGAGG |

PLA2G7, phospholipase A2 group VII; PLA2R1, phospholipase A2 receptor 1; PON1, paraoxonase-1; CERS4, ceramide synthase 4.

**Supplementary Table 2.** Anthropometrical and metabolic characteristics of nonobese and obese MASLD patients with/without liver biopsy.

|  | | **Without biopsy** | | | **With biopsy** | | |  |  |
| --- | --- | --- | --- | --- | --- | --- | --- | --- | --- |
|  | **Nonobese MASLD** | | **Obese**  **MASLD** | | | **Nonobese MASLD** | **Obese**  **MASLD** |  |  |
| **Characteristics** | **(n = 148)** | | **(n = 124)** | | | **(n = 52)** | **(n = 76)** | ***P(1 vs 3)*** | ***P(2 vs 4)*** |
| Age, year | 32.8 ± 12.8 | | 41.5 ± 12.2 | 34.5 ± 13.7 | | | 40.1 ± 13.2 | 0.45 | 0.58 |
| Male, n (%) | 104 (70.3) | | 92 (74.2%) | 42 (80.8%) | | | 61 (80.3%) | 0.14 | 0.33 |
| BMI, kg/m^2^ | 22.3 ± 1.8 | | 28.9 ± 3.0 | 22.8 ± 1.0 | | | 29.6 ± 4.3 | 0.08 | 0.14 |
| Waist circumstance, cm | 83.2 ± 6.0 | | 95.1 ± 8.4 | 82.9 ± 4.9 | | | 95.6 ± 8.8 | 0.74 | 0.68 |
| Systolic blood pressure, mmHg | 127 ± 21 | | 131 ± 16 | 129 ± 14 | | | 133 ± 13 | 0.62 | 0.38 |
| Diastolic blood pressure, mmHg | 82 ± 13 | | 87 ± 12 | 82 ± 8 | | | 90 ± 12 | 0.72 | 0.21 |
| Alanine aminotransferase, U/L † | 51 (43-78) | | 71 (35-119) | 57 (40-90) | | | 76 (33-127) | 0.54 | 0.47 |
| Aspartate aminotransferase, U/L † | 31 (24-40) | | 50 (33-61) | 35 (28-45) | | | 54 (36-59) | 0.10 | 0.51 |
| Total cholesterol, mmol/L | 4.8 ± 1.0 | | 5.2 ± 1.0 | 4.8 ± 0.8 | | | 5.4 ± 1.1 | 0.79 | 0.39 |
| Triglycerides, mmol/L | 1.9 ± 1.2 | | 2.5 ± 1.0 | 2.1 ± 1.6 | | | 2.3 ± 0.9 | 0.43 | 0.81 |
| HDL- cholesterol, mmol/L | 1.3 ± 0.3 | | 1.9 ± 0.8 | 1.4 ± 0.4 | | | 1.7 ± 0.7 | 0.42 | 0.75 |
| LDL- cholesterol, mmol/L | 3.2 ± 0.7 | | 3.6 ± 0.7 | 3.1 ± 0.7 | | | 3.4 ± 0.8 | 0.82 | 0.71 |
| Fasting blood glucose, mmol/L | 5.1 ± 1.0 | | 5.0 ± 1.1 | 5.0 ± 1.0 | | | 5.1 ± 1.3 | 0.22 | 0.56 |
| Fasting insulin, μU/mL † | 8.9 (6.7-11.2) | | 12.1 (8.1-21.9) | 9.5 (8.0-11.5) | | | 14.1 (9.9-23.4) | 0.12 | 0.29 |
| HOMA-IR† | 1.8 (1.2-2.6) | | 2.3 (1.2-3.7) | 1.9 (1.2-3.6) | | | 2.5 (1.4-3.4) | 0.09 | 0.34 |
| HOMA-IR > 2.5, n (%) | 38 (25.7) | | 56 (45.2) | 14 (26.9) | | | 38 (50.0) | 0.86 | 0.51 |
| Uric acid, μmol/L | 383 ± 93 | | 428 ± 114 | 406 ± 82 | | | 443 ± 121 | 0.12 | 0.24 |
| Liver fat content, %† | 12.5 (7.8-19.0) | | 14.5 (10.0-23.1) | 16.1 (7.1-20.1) | | | 18.4 (9.6-28.7) | 0.88 | 0.12 |
| Liver stiffness, kPa | 6.3 ± 3.5 | | 6.7 ± 3.4 | 5.8 ± 1.5 | | | 7.0 ± 2.3 | 0.32 | 0.54 |
| Steatosis grade |  | |  |  | | |  | 0.12 |  |
| 1 | - | | - | 23(44.2) | | | 34(44.7) |  |  |
| 2 | - | | - | 25(48.1) | | | 27(51.9) |  |  |
| 3 | - | | - | 4(7.7) | | | 15(19.7) |  |  |
| Lobular inflammation |  | |  |  | | |  | 0.001 |  |
| 0 | - | | - | 8(15.4) | | | 5(6.6) |  |  |
| 1 | - | | - | 44(84.6) | | | 56(73.7) |  |  |
| 2 | - | | - | 0(0) | | | 15(19.7) |  |  |
| Ballooning |  | |  |  | | |  | 0.09 |  |
| 0 | - | | - | 34(65.4) | | | 37(48.7) |  |  |
| 1 | - | | - | 18(34.6) | | | 36(47.4) |  |  |
| 2 | - | | - | 0(0) | | | 3(3.9) |  |  |
| Fibrosis stage |  | |  |  | | |  | <0.001 |  |
| 0 | - | | - | 40(76.9) | | | 28(36.8) |  |  |
| 1 | - | | - | 9(17.3) | | | 36(47.4) |  |  |
| 2 | - | | - | 3(5.8) | | | 10(13.2) |  |  |
| 3 | - | | - | 0(0) | | | 1(1.3) |  |  |
| 4 | - | | - | 0(0) | | | 1(1.3) |  |  |
| MASLD activity score (NAS) | - | | - | 3 (2-4) | | | 3 (2-4) | 0.012 |  |
| SAF score | - | | - | 3 (2-4) | | | 4 (2-5) | 0.002 |  |
| Steatohepatitis, n (%) | - | | - | 12 (23.1) | | | 39 (51.3) | 0.001 |  |

BMI, body mass index; HDL-cholesterol, high-density lipoprotein-cholesterol; LDL-cholesterol, low-density lipoprotein-cholesterol; HOMA-IR, homeostasis model assessment of insulin resistance.

**Supplementary Table 3.** Anthropometrical and metabolic characteristics of nonobese and obese MASLD patients with metabonomics.

|  | **Normal Controls** | **Nonobese MASLD** | **Obese MASLD** |  |
| --- | --- | --- | --- | --- |
| **Characteristics** | **(n = 80)** | **(n = 80)** | **(n = 80)** | ***P*** |
| Age, year | 42.0 ± 14.0 | 45.3 ± 13.6 | 43.5 ± 12.8 | 0.23 |
| Male, n (%) | 52 (65.0%) | 53 (66.3%) | 60 (75.0%) | 0.39 |
| BMI, kg/m^2^ | 22.1 ± 3.3 | 23.5 ± 1.8 | 29.4 ± 3.0 | <0.001^a,b,c^ |
| Waist circumstance, cm | 78.3 ± 10.1 | 82.9 ± 6.2 | 94.8 ± 7.9 | <0.001 ^a,b,c^ |
| Systolic blood pressure, mmHg | 121 ± 16 | 128 ± 20 | 131 ± 19 | 0.74 |
| Diastolic blood pressure, mmHg | 78 ± 12 | 83 ± 12 | 87 ± 13 | 0.52 |
| Alanine aminotransferase, U/L † | 24 (18-39) | 42 (24-68) | 56 (29-98) | <0.001 ^a,b^ |
| Aspartate aminotransferase, U/L † | 26 (21-40) | 31 (23-43) | 37 (23-50.8) | 0.07 |
| Total cholesterol, mmol/L | 5.0 ± 1.5 | 5.3 ± 1.2 | 5.3 ± 1.1 | 0.38 |
| Triglycerides, mmol/L | 1.2 ± 0.9 | 2.1 ± 1.6 | 2.1 ± 1.6 | 0.043 ^a,b^ |
| HDL- cholesterol, mmol/L | 1.3 ± 0.3 | 1.3 ± 0.8 | 1.3 ± 0.7 | 0.90 |
| LDL- cholesterol, mmol/L | 2.9 ± 0.7 | 3.3 ± 0.8 | 3.3 ± 0.7 | 0.026 ^a,b^ |
| Fasting blood glucose, mmol/L | 4.7 ± 1.1 | 5.3 ± 1.1 | 5.0 ± 0.9 | 0.02 ^a^ |
| Fasting insulin, μU/mL † | 9.6 (3.9-10.2) | 8.6 (6.0-11.6) | 11.3 (8.6-18.5) | <0.001 ^b,c^ |
| HOMA-IR† | 2.0 (0.9-2.3) | 2.0 (1.3-2.8) | 2.5 (1.8-4.1) | <0.001 ^b,c^ |
| HOMA-IR > 2.5, n (%) | 4 (5.0%) | 20 (25.0%) | 35 (43.8%) | <0.001 ^b,c^ |
| Uric acid, μmol/L | 372 ± 101 | 388 ± 107 | 432 ± 111 | 0.04 ^b,c^ |
| Liver fat content, %† | 3.9 (3.2-4.3) | 12.7 (6.9-19.8) | 15.4 (10.9-24.5) | <0.001 ^a,b,c^ |
| Liver stiffness, kPa | 6.0 ± 1.3 | 6.3 ± 2.1 | 6.2 ± 1.8 | 0.86 |

BMI, body mass index; HDL-cholesterol, high-density lipoprotein-cholesterol; LDL-cholesterol, low-density lipoprotein-cholesterol; HOMA-IR, homeostasis model assessment of insulin resistance.

**Supplementary Table 4** Characteristics of the study cohort based on the PLA2R rs35771982 genotype.

|  | **AA** | **AT** | **TT** |  |
| --- | --- | --- | --- | --- |
| **Characteristics** | **(n = 544)** | **(n = 42)** | **(n = 14)** | ***P*** |
| Age, year | 41.7 ± 12.9 | 40.9 ± 10.4 | 42.4 ± 11.7 | 0.52 |
| Male, n (%) | 374 (70.8%) | 26 (63.4%) | 10 (71.4%) | 0.60 |
| BMI, kg/m^2^ | 24.9 ± 3.96 | 24.5 ± 3.6 | 21.8 ± 3.8 | 0.014^bc^ |
| Waist circumstance, cm | 86.8 ± 9.9 | 82.9 ± 6.9 | 91.0 ± 11.3 | 0.10 |
| Systolic blood pressure, mmHg | 126.7 ± 18.5 | 120.3 ± 21.0 | 121.7 ± 17.0 | 0.08 |
| Diastolic blood pressure, mmHg | 82.6 ± 12.8 | 78.3 ± 16.3 | 75.2 ± 10.6 | 0.025^ab^ |
| Alanine aminotransferase, U/L † | 31 (20-57) | 35 (20-56) | 26 (25-40) | 0.08 |
| Aspartate aminotransferase, U/L † | 28 (23-40) | 24 (20-40) | 25 (21-27) | 0.13 |
| Total cholesterol, mmol/L | 5.2 ± 1.1 | 5.1 ± 1.2 | 4.8 ± 0.4 | 0.83 |
| Triglycerides, mmol/L | 1.7 ± 1.4 | 1.5 ± 0.8 | 1.0 ± 0.5 | 0.71 |
| HDL- cholesterol, mmol/L | 1.3 ± 0.5 | 1.3 ± 0.2 | 1.3 ± 0.3 | 0.94 |
| LDL- cholesterol, mmol/L | 3.2 ± 0.9 | 3.1 ± 0.7 | 2.9 ± 0.4 | 0.89 |
| Fasting blood glucose, mmol/L | 5.1 ± 0.9 | 4.9 ± 1.4 | 4.6 ± 0.3 | 0.26 |
| Fasting insulin, μU/mL † | 8.3 (5.1-11.8) | 7.2 (5.2-10.8) | 4.0 (2.9-5.3) | 0.008^abc^ |
| HOMA-IR† | 1.9 (1.1-2.7) | 1.5 (1.1-2.9) | 0.8 (0.6-1.2) | 0.28 |
| Uric acid, μmol/L† | 377 (305-445) | 352 (300-434) | 299 (207-384) | 0.06 |
| Liver fat content, %† | 15.9 (8.7-24.5) | 10.7 (7.2-18.6) | 8.2 (4.5-15.3) | <0.001^abc^ |
| Liver stiffness, kPa | 6.3 ± 3.0 | 6.3 ± 1.8 | 4.9 ± 0.8 | 0.80 |

^a^*P* < 0.05 between AA genotype and AT genotype; ^b^*P* < 0.05 between AA genotype and TT genotype;

^c^*P* < 0.05 between AT genotype and TT genotype.

**Supplementary Table 5** Characteristics of the study cohort based on the PLA2R rs35771982 genotype.

|  | **GG** | **GC** | **CC** |  |
| --- | --- | --- | --- | --- |
| **Characteristics** | **(n = 312)** | **(n = 237)** | **(n = 51)** | ***P*** |
| Age, year | 42.2 ± 12.8 | 40.1 ± 12.6 | 43.8 ± 11.5 | 0.08 |
| Male, n (%) | 224 (73.7%) | 156 (67.5%) | 30 (62.5%) | 0.14 |
| BMI, kg/m^2^ | 24.5 ± 3.5 | 24.9 ± 4.2 | 25.3 ± 3.6 | 0.34 |
| Waist circumstance, cm | 86.8 ± 8.3 | 85.9 ± 11.6 | 87.7 ± 9.0 | 0.48 |
| Systolic blood pressure, mmHg | 125.5 ± 20.5 | 125.6 ± 16.5 | 131.5 ± 16.3 | 0.14 |
| Diastolic blood pressure, mmHg | 81.3 ± 12.7 | 82.0 ± 13.8 | 87.2 ± 11.2 | 0.025^bc^ |
| Alanine aminotransferase, U/L † | 31 (19-56) | 32 (21-57) | 39 (21-62) | 0.29 |
| Aspartate aminotransferase, U/L † | 28 (22-38) | 29 (23-40) | 28 (24-40) | 0.39 |
| Total cholesterol, mmol/L | 5.3 ± 1.2 | 5.2 ± 1.0 | 5.5 ± 1.1 | 0.83 |
| Triglycerides, mmol/L | 1.7 ± 0.9 | 1.4 ± 0.8 | 1.5 ± 0.5 | 0.71 |
| HDL- cholesterol, mmol/L | 1.3 ± 0.5 | 1.3 ± 0.3 | 1.3 ± 0.3 | 0.51 |
| LDL- cholesterol, mmol/L | 3.3 ± 0.9 | 3.3 ± 0.8 | 3.4 ± 0.9 | 0.62 |
| Fasting blood glucose, mmol/L | 5.0 ± 0.8 | 5.0 ± 1.0 | 5.3 ± 1.2 | 0.12 |
| Fasting insulin, μU/mL † | 8.4 (5.3-11.8) | 8.1 (5.9-11.7) | 8.5 (6.5-10.9) | 0.95 |
| HOMA-IR† | 1.7 (1.1-2.5) | 1.9 (1.3-2.8) | 1.9 (1.2-3.1) | 0.08 |
| Uric acid, μmol/L† | 372 (312-434) | 374 (321-432) | 351 (303-420) | 0.35 |
| Liver fat content, %† | 11.4 (6.8-18.4) | 11.9 (6.2-21.2) | 10.9 (5.0-16.8) | 0.66 |
| Liver stiffness, kPa | 6.3 ± 3.1 | 6.2 ± 2.9 | 6.2 ± 1.6 | 0.95 |

^a^*P* < 0.05 between GG genotype and GC genotype; ^b^*P* < 0.05 between GG genotype and CC genotype;

^c^*P* < 0.05 between GC genotype and CC genotype.

**Supplementary Table 6** Characteristics of the study cohort based on the PLA2R rs3749117 genotype.

|  | **TT** | **CT** | **CC** |  |
| --- | --- | --- | --- | --- |
| **Characteristics** | **(n = 311)** | **(n = 238)** | **(n = 51)** | ***P*** |
| Age, year | 42.0 ± 13.0 | 40.4 ± 12.5 | 43.7 ± 11.1 | 0.16 |
| Male, n (%) | 217 (72.3%) | 161 (69.1%) | 32 (64.0%) | 0.43 |
| BMI, kg/m^2^ | 25.2 ± 3.9 | 24.3 ± 3.7 | 24.4 ± 3.4 | 0.024^a^ |
| Waist circumstance, cm | 88.4 ± 9.0 | 84.4 ± 10.6 | 85.1 ± 8.5 | 0.001^a^ |
| Systolic blood pressure, mmHg | 125.5 ± 20.3 | 125.8 ± 16.9 | 132.1 ± 16.1 | 0.08 |
| Diastolic blood pressure, mmHg | 82.0 ± 12.9 | 81.3 ± 13.8 | 85.9 ± 10.8 | 0.10 |
| Alanine aminotransferase, U/L † | 31 (19-57) | 33 (22-56) | 35 (21-56) | 0.59 |
| Aspartate aminotransferase, U/L † | 28 (22-40) | 30 (23-39) | 28 (23-37) | 0.49 |
| Total cholesterol, mmol/L | 5.3 ± 1.3 | 5.2 ± 1.0 | 5.5 ± 1.1 | 0.44 |
| Triglycerides, mmol/L | 1.7 ± 1.1 | 1.6 ± 1.0 | 1.7 ± 1.2 | 0.42 |
| HDL- cholesterol, mmol/L | 1.3 ± 0.5 | 1.3 ± 0.3 | 1.3 ± 0.3 | 0.65 |
| LDL- cholesterol, mmol/L | 3.3 ± 0.9 | 3.3 ± 0.8 | 3.4 ± 0.9 | 0.46 |
| Fasting blood glucose, mmol/L | 5.0 ± 0.8 | 5.0 ± 1.0 | 5.2 ± 1.2 | 0.36 |
| FINS, μU/mL † | 8.9 (5.6-12.1) | 7.9 (5.8-11.6) | 7.5 (5.9-10.2) | 0.41 |
| HOMA-IR† | 1.7 (1.2-2.6) | 1.9 (1.3-2.7) | 1.9 (1.2-3.0) | 0.40 |
| Uric acid, μmol/L† | 376 (314-438) | 372 (310-429) | 350 (304-431) | 0.37 |
| Liver fat content, %† | 11.5 (6.9-18.5) | 11.9 (6.2-20.5) | 11.7 (4.9-18.5) | 0.78 |
| Liver stiffness, kPa | 6.3 ± 3.0 | 6.2 ± 2.9 | 6.2 ± 1.7 | 0.94 |

^a^*P* < 0.05 between TT genotype and CT genotype; ^b^*P* < 0.05 between TT genotype and CC genotype;

^c^*P* < 0.05 between CT genotype and CC genotype.

**Supplementary Table 7** Characteristics of the study cohort based on the CERS4 rs17160348 genotype.

|  | **CC** | **CT** | **TT** |  |
| --- | --- | --- | --- | --- |
| **Characteristics** | **(n = 364)** | **(n = 104)** | **(n = 32)** | ***P*** |
| Age, year | 41.4 ± 12.7 | 42.3 ± 12.2 | 40.1 ± 13.5 | 0.66 |
| Male, n (%) | 254 (69.9) | 72 (68.8) | 26 (81.3) | 0.14 |
| BMI, kg/m^2^ | 24.8 ± 3.6 | 24.6 ± 4.1 | 25.0 ± 5.3 | 0.80 |
| Waist circumstance, cm | 85.7 ± 11.0 | 86.4 ± 9.2 | 92.0 ± 11.9 | 0.033^ab^ |
| Systolic blood pressure, mmHg | 126.1 ± 19.5 | 126.2 ± 14.3 | 125.2 ± 20.2 | 0.96 |
| Diastolic blood pressure, mmHg | 81.3 ± 12.7 | 82.0 ± 13.8 | 87.2 ± 11.2 | 0.41 |
| Alanine aminotransferase, U/L † | 33 (21-59) | 33 (21-59) | 25 (18-48) | 0.59 |
| Aspartate aminotransferase, U/L † | 29 (23-39) | 29 (23-39) | 25 (21-40) | 0.49 |
| Total cholesterol, mmol/L | 4.8 ± 0.9 | 5.3 ± 1.2 | 5.3 ± 1.1 | 0.042^ab^ |
| Triglycerides, mmol/L | 1.7 ± 1.1 | 1.6 ± 0.8 | 1.5 ± 0.9 | 0.32 |
| HDL- cholesterol, mmol/L | 1.3 ± 0.5 | 1.3 ± 0.3 | 1.2 ± 0.3 | 0.77 |
| LDL- cholesterol, mmol/L | 2.9 ± 0.6 | 3.3 ± 0.8 | 3.3 ± 0.9 | 0.034^ab^ |
| Fasting blood glucose, mmol/L | 5.1 ± 0.9 | 5.0 ± 1.0 | 4.9 ± 0.7 | 0.44 |
| Fasting insuli, μU/mL † | 8.7 (6.0-11.9) | 8.7 (6.0-11.9) | 6.7 (4.2-11.0) | 0.41 |
| HOMA-IR† | 1.8 (1.3-2.7) | 1.8 (1.3-2.7) | 1.4 (0.9-2.2) | 0.40 |
| Uric acid, μmol/L† | 377 (313-435) | 377 (314-435) | 340 (271-452) | 0.37 |
| Liver fat content, %† | 12.1 (6.5-20.1) | 12.1 (6.5-20.1) | 9.7 (4.7-23.5) | 0.78 |
| Liver stiffness, kPa | 6.3 ± 3.1 | 6.2 ± 2.9 | 6.4 ± 2.7 | 0.35 |

^a^*P* < 0.05 between CC genotype and CT genotype; ^b^*P* < 0.05 between CC genotype and TT genotype;

^c^*P* < 0.05 between CT genotype and TT genotype.

**Supplementary Table 8** Factors associated with moderate-severe steatosis, steatohepatitis and significant fibrosis in nonobese MASLD patients.

| Genotype | Moderate-to-severe steatosis | |  | Steatohepatitis | |  | Significant fibrosis | |
| --- | --- | --- | --- | --- | --- | --- | --- | --- |
|  | OR (95%CI) | *P* |  | OR (95%CI) | *P* |  | OR (95%CI) | *P* |
| PON1 rs854560 |  |  |  |  |  |  |  |  |
| AA | Ref. |  |  | Ref. |  |  | Ref. |  |
| AT+TT | 2.07 (0.58-7.44) | 0.26 |  | 1.35 (0.40-4.48) | 0.63 |  | 1.25 (0.84-2.06) | 0.41 |
| PLA2R1 rs35771982 |  |  |  |  |  |  |  |  |
| GG | Ref. |  |  | Ref. |  |  | Ref. |  |
| GC+CC | 0.78 (0.23-2.65) | 0.70 |  | 2.61 (1.15-3.87) | 0.028 |  | 1.12 (0.33-3.82) | 0.86 |
| PLA2R1 rs3749117 |  |  |  |  |  |  |  |  |
| TT | Ref. |  |  | Ref. |  |  | Ref. |  |
| TC+CC | 2.17 (0.52-9.1) | 0.29 |  | 1.34 (0.50-4.02) | 0.59 |  | 0.64 (0.16-2.58) | 0.53 |
| CERS4 rs17160348 |  |  |  |  |  |  |  |  |
| CC | Ref. |  |  | Ref. |  |  | Ref. |  |
| CT+TT | 2.20 (0.65-7.48) | 0.21 |  | 1.61 (0.49-5.26) | 0.43 |  | 1.22 (0.39-3.80) | 0.73 |

PLA2R1, phospholipase A2 receptor 1; PON1, paraoxonase-1; CERS4, ceramide synthase 4.

**Supplementary Table 9 Factors associated with moderate-severe steatosis, steatohepatitis and significant fibrosis in obese MASLD patients.**

| **Genotype** | **Moderate-to-severe steatosis** | |  | **Steatohepatitis** | |  | **Significant fibrosis** | |
| --- | --- | --- | --- | --- | --- | --- | --- | --- |
|  | **OR (95%CI)** | ***P*** |  | **OR (95%CI)** | ***P*** |  | **OR (95%CI)** | ***P*** |
| **PON1 rs854560** |  |  |  |  |  |  |  |  |
| AA | Ref. |  |  | Ref. |  |  | Ref. |  |
| AT+TT | 1.67 (0.54-5.01) | 0.38 |  | 0.38 (0.12-1.20) | 0.10 |  | 1.19 (0.28-4.49) | 0.81 |
| **PLA2R1 rs35771982** |  |  |  |  |  |  |  |  |
| GG | Ref. |  |  | Ref. |  |  | Ref. |  |
| GC+CC | 0.89 (0.36-2.20) | 0.80 |  | 0.40 (0.14-1.11) | 0.08 |  | 0.94 (0.27-3.22) | 0.91 |
| **PLA2R1 rs3749117** |  |  |  |  |  |  |  |  |
| TT | Ref. |  |  | Ref. |  |  | Ref. |  |
| TC+CC | 2.59 (0.88-7.67) | 0.10 |  | 1.32 (1.12-1.83) | 0.019 |  | 0.85 (0.21-3.51) | 0.82 |
| **CERS4 rs17160348** |  |  |  |  |  |  |  |  |
| CC | Ref. |  |  | Ref. |  |  | Ref. |  |
| CT+TT | 1.27 (0.40-4.01) | 0.68 |  | 0.38 (0.12-1.20) | 0.10 |  | 2.76 (0.78-9.75) | 0.11 |

PLA2R1, phospholipase A2 receptor 1; PON1, paraoxonase-1; CERS4, ceramide synthase 4.

**Supplementary Figure**

**
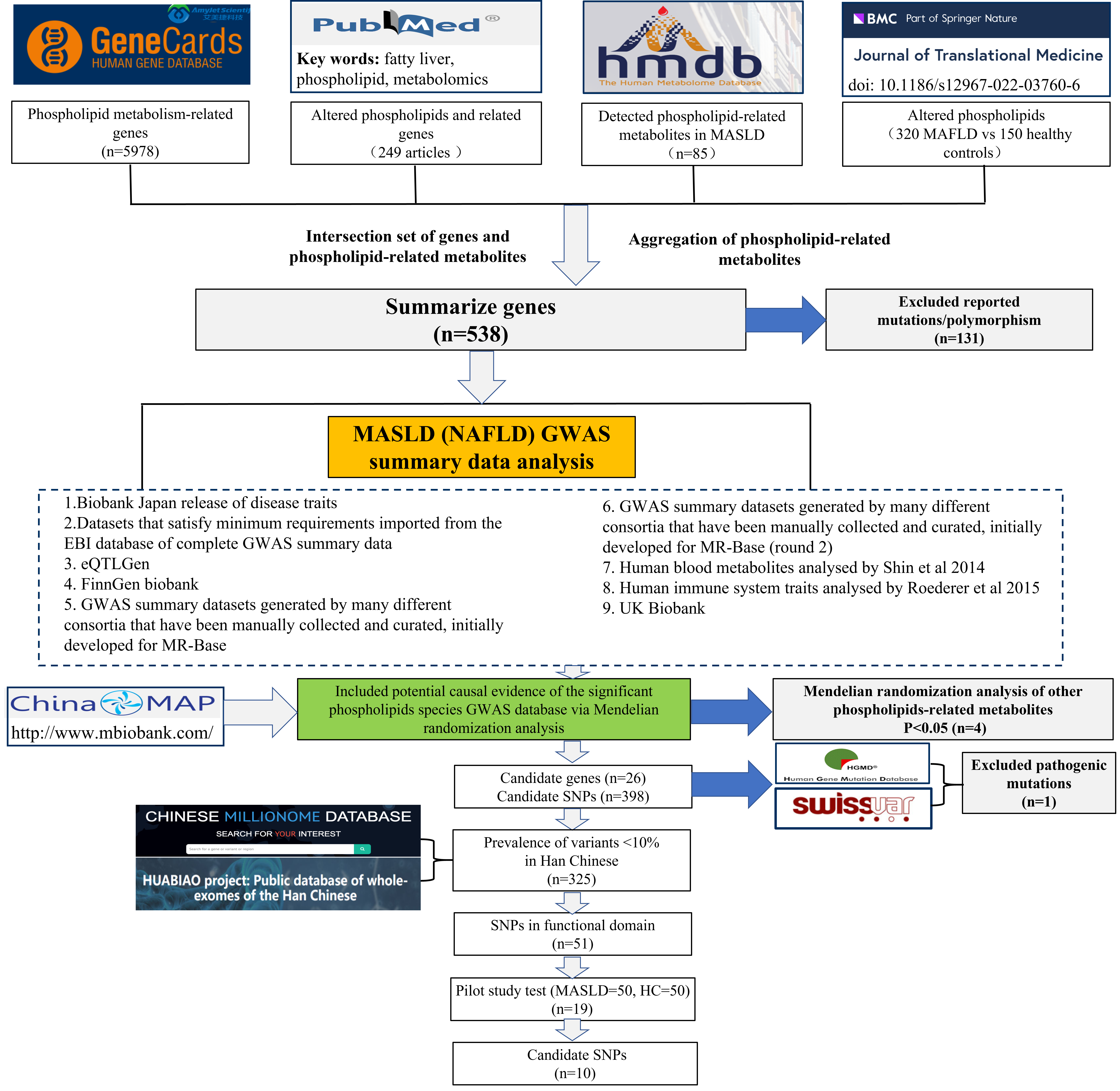
**

**Supplementary Figure 1.** Flowchart for the genes/variants selection process. We searched the GeneCards database, PubMed, HMDB, and our previous paper about metabolism changes in MASLD to identify altered changes of the phospholipid metabolism-related genes, altered phospholipids and related genes, phospholipid-related metabolites in MASLD and altered phospholipids. And we further chose the intersection set of genes and phospholipid-related metabolites and conducted the MASLD (NAFLD) genome-wide association (GWAS) summary data analysis to include potential causal evidence of the significant phospholipids species GWAS database via Mendelian randomization analysis. Therefore, we have the candidate genes and SNPs. We focused on the SNPs with the prevalence of variants <10% in Han Chinese, located in functional domain of proteins, and performed the pilot study test to identify the final 10 SNPs in the present study.

**
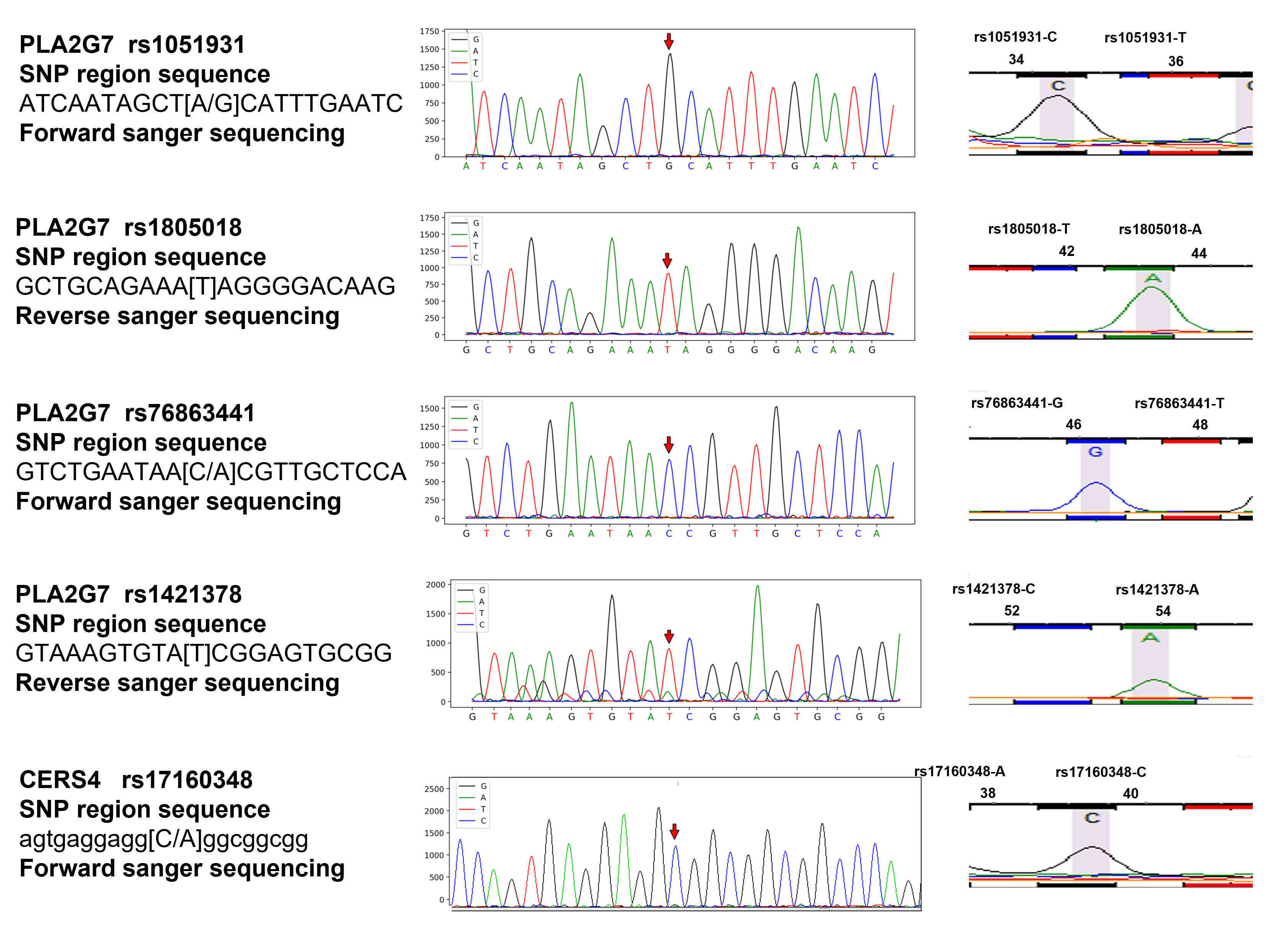
**

**A**

**B**

**C**

**D**

**E**

**F**


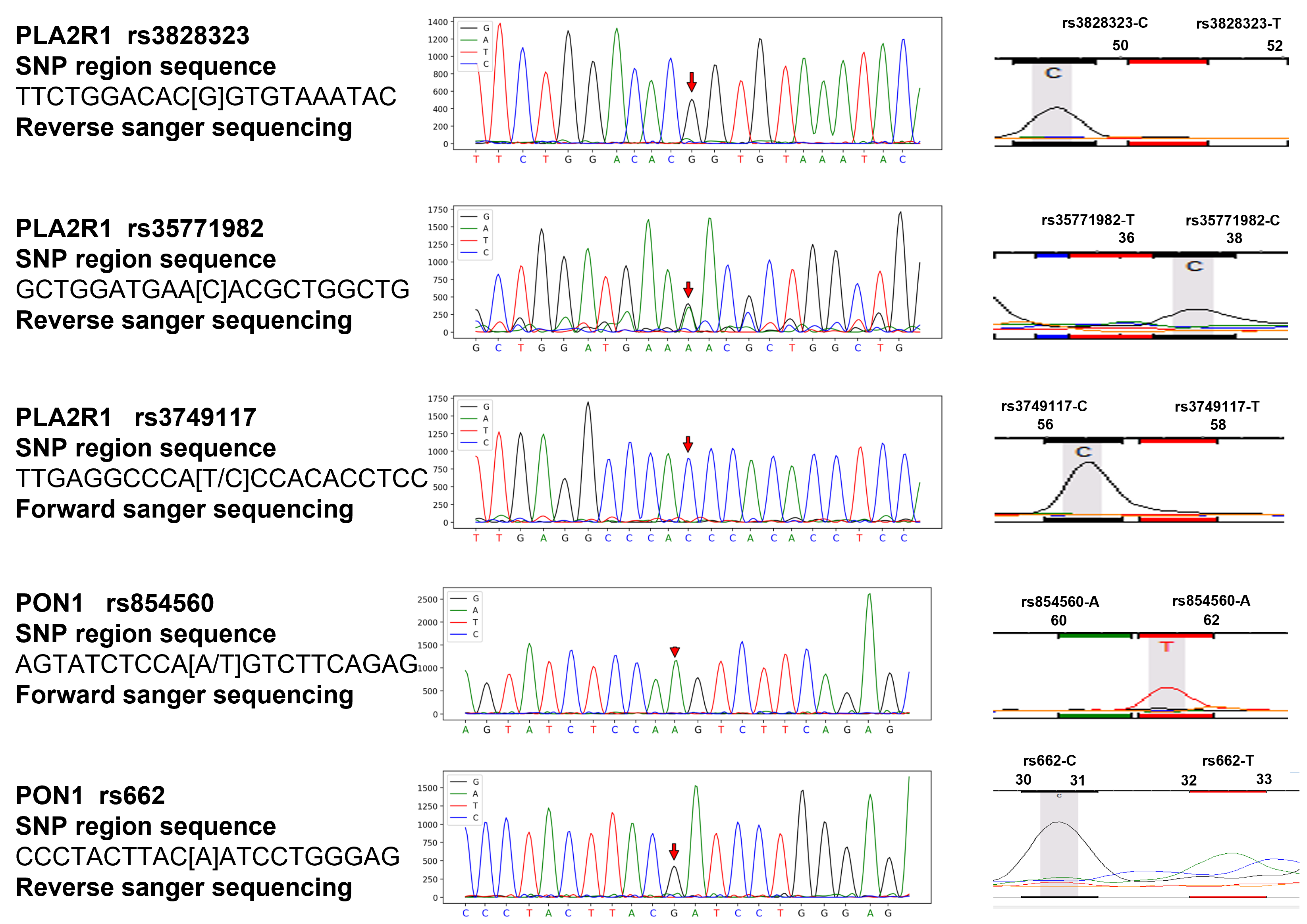


**G**

**H**

**I**

**J**

**Supplementary Figure 2.** The sequencing validation of SNaPshot method in SNP genotyping of 10 loci (PLA2G7 rs1805018, rs76863441, rs1421378, rs1051931; PLA2R1 rs35771982, rs3828323, rs3749117; PON1 rs662, rs854560; CERS4 rs17160348).

**Output results for Mendelian randomization analysis via R studio**

**Inputs**

library(TwoSampleMR)

library(MRInstruments)

library(ieugwasr)

test_exposure=read.table("PLA2 7 SNPs summary data",header = T,stringsAsFactors = F)

test_outcome=read.table("7 SNPs summary data",header = T,stringsAsFactors = F)

colnames(test_exposure)=c("chr","SNP","ps","n_miss","effect_allele","other_allele","eaf","beta","se","P")

test_exposure$Phenotype=exposure.name

dat_exp <- format_data(test_exposure, type="exposure")

colnames(test_outcome)=c("SNP","effect_allele","other_allele","eaf","beta","se","P")

test_outcome$Phenotype=outcome.name

dat_out <- format_data(test_outcome, type="outcome")

# harmonize

dat_harm <- harmonise_data(

exposure_dat = dat_exp,

outcome_dat = dat_out

)

dat_harm$mr_keep="TRUE"

dat_harm$mr_keep=as.logical(dat_harm$mr_keep)

# run analysis

test_MR <- mr(dat_harm,method_list=c("mr_egger_regression", "mr_ivw","mr_wald_ratio","mr_weighted_median"))

test_hetero=mr_heterogeneity(dat_harm)

test_pleiotropy=mr_pleiotropy_test(dat_harm)

write.table(test_MR,file = paste(outcome.name,exposure.name,"MR.txt",sep = "."),sep = "\t",quote = F,row.names = F)

write.table(test_hetero,file = paste(outcome.name,exposure.name,"heterogeneity.txt",sep = "."),sep = "\t",quote = F,row.names = F)

write.table(test_pleiotropy,file = paste(outcome.name,exposure.name,"pleiotropy.txt",sep = "."),sep = "\t",quote = F,row.names = F)

**Outputs**

**OutToExp.FattyLiver.PLA2.MR**

"id.exposure" "id.outcome" "outcome" "exposure" "method" "nsnp" "b" "se" "pval"

"1" "kmX6EA" "ebi-a-GCST90091033" "FattyLiver" "PLA2" "MR Egger" 5 -1.66264674668296 1.77664727840641 0.41839457405119

"2" "kmX6EA" "ebi-a-GCST90091033" "FattyLiver" "PLA2" "Inverse variance weighted" 5 0.539649802574027 0.30092538384089 0.0729248018616706

"3" "kmX6EA" "ebi-a-GCST90091033" "FattyLiver" "PLA2" "Weighted median" 5 0.576799911509478 0.35822745395001 0.107365128118807

**OutToExp.FattyLiver.PLA2.F.test**

"Exposure" "F value"

"1" "PLA2" "47.610306122449"

**OutToExp.FattyLiver.PLA2.Final.used.SNP.test**

"SNP" "effect_allele.exposure" "other_allele.exposure" "effect_allele.outcome" "other_allele.outcome" "beta.exposure" "beta.outcome" "eaf.exposure" "eaf.outcome" "remove" "palindromic" "ambiguous" "id.outcome" "chr.outcome" "pos.outcome" "se.outcome" "samplesize.outcome" "pval.outcome" "outcome" "mr_keep.outcome" "pval_origin.outcome" "chr.exposure" "se.exposure" "exposure" "mr_keep.exposure" "pval.exposure" "pval_origin.exposure" "id.exposure" "action" "SNP_index" "mr_keep"

"1" "rs10846744" "C" "G" "C" "G" 0.029 0.0148504 NA 0.16998 FALSE TRUE TRUE "ebi-a-GCST90091033" "12" "125312425" 0.0230766 778614 0.5199 "FattyLiver" TRUE "reported" 12 0.005 "PLA2" TRUE 6.63149195665233e-09 "inferred" "kmX6EA" 2 1 TRUE

"2" "rs3799277" "A" "G" "A" "G" 0.027 0.0295777 NA 0.237575 FALSE FALSE FALSE "ebi-a-GCST90091033" "6" "46657057" 0.0212604 778614 0.1642 "FattyLiver" TRUE "reported" 6 0.004 "PLA2" TRUE 1.47845155560356e-11 "inferred" "kmX6EA" 2 1 TRUE

"5" "rs6511720" "T" "G" "T" "G" -0.045 0.0059845 NA 0.110338 FALSE FALSE FALSE "ebi-a-GCST90091033" "19" "11202306" 0.0271904 778614 0.8258 "FattyLiver" TRUE "reported" 19 0.007 "PLA2" TRUE 1.28808723502674e-10 "inferred" "kmX6EA" 2 1 TRUE

"6" "rs7528419" "A" "G" "A" "G" 0.035 0.0207689 NA 0.787276 FALSE FALSE FALSE "ebi-a-GCST90091033" "1" "109817192" 0.0193844 778614 0.284 "FattyLiver" TRUE "reported" 1 0.004 "PLA2" TRUE 2.13352747509497e-18 "inferred" "kmX6EA" 2 1 TRUE

"7" "rs964184" "C" "G" "C" "G" -0.032 -0.0313401 NA 0.837972 FALSE TRUE TRUE "ebi-a-GCST90091033" "11" "116648917" 0.0235161 778614 0.1826 "FattyLiver" TRUE "reported" 11 0.005 "PLA2" TRUE 1.55376951634196e-10 "inferred" "kmX6EA" 2 1 TRUE

**OutToExp.FattyLiver.PLA2.heterogeneity.test**

"id.exposure" "id.outcome" "outcome" "exposure" "method" "Q" "Q_df" "Q_pval"

"1" "kmX6EA" "ebi-a-GCST90091033" "FattyLiver" "PLA2" "MR Egger" 0.524223836813069 3 0.913538206188498

"2" "kmX6EA" "ebi-a-GCST90091033" "FattyLiver" "PLA2" "Inverse variance weighted" 2.10616691975271 4 0.716239500518404

**OutToExp.FattyLiver.PLA2.Outliers.test**

"x"

"1" "rs4420638"

"2" "rs4420638"

"3" "rs445925"

**OutToExp.FattyLiver.PLA2.pleiotropy.test**

"id.exposure" "id.outcome" "outcome" "exposure" "egger_intercept" "se" "pval"

"1" "kmX6EA" "ebi-a-GCST90091033" "FattyLiver" "PLA2" 0.0745844712690549 0.0592997672013186 0.297475766758448
